# Reciprocal relationships between parental and scholastic homework assistance and students’ academic functioning at elementary school

**DOI:** 10.3389/fpsyg.2023.1106362

**Published:** 2023-04-28

**Authors:** Lisa Benckwitz, Katharina Kohl, Janina Roloff, Oliver Lüdtke, Karin Guill

**Affiliations:** IPN – Leibniz Institute for Science and Mathematics Education, Kiel, Germany

**Keywords:** parental homework assistance, homework assistance at school, homework behavior, academic functioning, academic achievement, elementary school

## Abstract

Homework assistance is provided both by parents and by institutions, for example, full-day schools. Previous research found evidence that the quality of homework assistance – measured by three dimensions derived from the self-determination theory, namely, responsiveness, structure, and control – is reciprocally related with students’ academic functioning (i.e., achievement and homework behavior). However, findings on parental homework assistance have been consistent only for the secondary level, whereas elementary school students have been studied less and previous results obtained for this population are inconclusive. Moreover, research on homework assistance that is given at school is scarce. Therefore, the present study aimed to investigate whether reciprocal associations between the quality of homework assistance and students’ academic functioning in elementary school can be found (1) for parental homework assistance and (2) for scholastic homework assistance. We calculated cross-lagged panel models based on longitudinal data from 335 German fourth graders collected in autumn 2019 (September and October) and winter 2020 (February and March). The analyses for scholastic homework assistance were based on a subsample of 112 students. Whereas responsiveness and structure did not predict students’ outcomes in the way we expected, control had unfavorable relationships in both homework settings. Moreover, parental control was reciprocally related with students’ mean grades in three subjects. The positive forms of homework assistance (responsiveness and structure) were predicted by different indicators of academic functioning in the two homework settings.

## 1. Introduction

Parents can be involved in their children’s school career in various ways, for example, in school-to-home communication, involvement at school, and involvement in learning activities at home (Epstein, 1986). Although there has been discussion about the justification of homework due to the high costs for children, parents, and teachers (e.g., time-related costs or arguments within the family; Trautwein et al., 2006; Dumont et al., 2012; Núñez et al., 2015), homework is an integral part of most students’ daily life (Cooper et al., 2006). Moreover, previous research has found largely positive effects of the completion of homework tasks on students’ academic achievement (Cooper et al., 2006; Fan et al., 2017). Thus, parental homework assistance can be considered to be a form of parental involvement that is both very common and important for children’s educational careers (Patall et al., 2008). At the same time, homework assistance is now often taken over or supported by institutions, for example, full-day schools. The importance of scholastic homework assistance in Germany has grown due to the demand for the compatibility of family and work life and also the demand to provide better learning conditions for all students in the context of social inequalities in education (Nordt, 2020; Sauerwein and Rother, 2022). Moreover, scholastic homework assistance has the potential to relieve families because parental homework assistance often leads to conflicts between parents and children (Dumont et al., 2012).

Prior quantitative research on the relationship of homework assistance with students’ outcomes focused almost exclusively on parental homework assistance, and literature on scholastic homework assistance is scarce. Regarding parental homework assistance, previous studies found that it can be both beneficial for and detrimental to students’ academic development (Patall et al., 2008; Wilder, 2014; Xu et al., 2018). This suggests that parental homework assistance is not *per se* a positive resource but that its effects depend on specific circumstances. Along these lines, studies that focused on the quality of parental homework assistance instead of, or in addition to, the quantity showed that the quality is more relevant for students’ academic functioning (i.e., achievement and homework behavior; Dumont et al., 2014) than the quantity. These studies described high-quality homework assistance as being characterized by responsiveness, structure, emotional support, and autonomy support, whereas controlling and intrusive parental behavior represents low-quality assistance (Dumont et al., 2014; Moroni et al., 2015; Guill et al., 2020). Prior research provided evidence for bidirectional links, with students’ academic functioning being both the outcome and the antecedent of parental homework assistance (e.g., Niggli et al., 2007; Dumont et al., 2014; Núñez et al., 2017; Guill et al., 2020). This picture has been consistently shown in studies at the secondary level, with multiple studies reporting relationships between the quality of parental homework assistance, students’ academic achievement (Niggli et al., 2007; Dumont et al., 2014; Moroni et al., 2015; Núñez et al., 2015), and students’ homework behavior (Trautwein et al., 2006; Dumont et al., 2014; Núñez et al., 2015; Guill et al., 2020). However, at the elementary level, the pattern of results is less clear because less research has been conducted and the results varied across studies, across quality indicators of parental homework assistance, and across indicators of students’ academic functioning (e.g., Silinskas et al., 2013; Núñez et al., 2015; Cunha et al., 2018).

Due to the inconsistency of the results of previous research at the elementary level, in the present study, we examined whether similar bidirectional links between homework assistance and students’ academic functioning that have been found in secondary school can also be found for a sample of elementary school children in Germany. On the basis of the empirical evidence outlined above, we focused on the quality of parental homework assistance and included quality dimensions that have been found to be relevant in secondary school samples, namely, responsiveness, structure, and control (Dumont et al., 2014). In addition, with research on scholastic homework assistance being scarce, we examined these links not only for parental but also for scholastic homework assistance.

### 1.1. The quality of parental homework assistance

Research on the quality of homework assistance (Nordt, 2013; Dumont et al., 2014; Guill et al., 2020) often draws on the self-determination theory (SDT; Ryan and Deci, 2000). The SDT concerns the role that contextual conditions play in motivational and personality development, which makes it a valuable theory to investigate the role of parental and scholastic homework assistance in students’ academic functioning. According to the SDT, students’ intrinsic motivation, personal growth, and well-being are promoted in learning environments that allow the satisfaction of three innate psychological needs, namely, competence, social relatedness, and autonomy (Deci et al., 1991; Ryan and Deci, 2000). On the basis of the SDT, Dumont et al. (2014) proposed three quality dimensions of parental homework assistance: *responsiveness*, *structure*, and *control*. To measure the quality of parental homework assistance, student reports are commonly used because they offer several benefits (for an overview of the benefits, see Dumont et al., 2014). For example, it has been argued that the child’s perception of the parental involvement forms the way in which the child responds to it (Hoover-Dempsey et al., 2005).

*Responsiveness* refers to being approachable and supportive during the homework process. For instance, it is characterized by helping the child when they express the need for help, listening to the child’s thoughts, and supporting them to overcome obstacles during homework preparation. By showing interest and supportive involvement in the homework of their child, parents can support students’ need for social relatedness (Dumont et al., 2014; Guill et al., 2020). Moreover, students’ autonomy is supported because the assistance is only provided at the request of the child. Previous research at the secondary level found that homework assistance that was perceived as responsive and supportive was positively related with students’ achievement, homework time management, homework effort, and expectancy to be able to master their homework; it was negatively related with homework procrastination (Trautwein et al., 2006; Dumont et al., 2014; Moroni et al., 2015; Núñez et al., 2015; Guill et al., 2020).

*Structure* during homework assistance refers to setting clear rules and guidelines for homework preparation, for example, by setting the rule that a student has to finish the homework first before meeting friends (Dumont et al., 2014; Guill et al., 2020). Providing a structured learning environment allows students to increase both the time they spend on learning and their learning effectiveness (Carroll, 1963). Therefore, it has the potential to positively affect students’ achievement and enhance their competency experience. In previous studies at the secondary level, parental homework assistance that was characterized by high structure was positively associated with students’ effort, but no associations with achievement were found (Dumont et al., 2014; Guill et al., 2020).

The third quality dimension, namely, *control*, is characterized by parents interfering without being asked for help or by parents threatening their children with punishment if they do not do their homework properly. It is theoretically assumed to negatively impact students’ academic functioning because it undermines their sense of competence and autonomy, by suggesting to the child that they are not able to solve the tasks independently, and their social relatedness, by creating a situation in which the child feels uncomfortable. Control is distinct from structure because it represents decidedly negative aspects of parental behavior, whereas structure refers to positive aspects of parental controlling behavior (Grolnick and Pomerantz, 2009; Dumont et al., 2014; Guill et al., 2020). In contrast to responsive and structuring homework assistance, controlling and intrusive behavior during the homework process showed unfavorable relationships with students’ academic functioning at the secondary level, for example, with lower academic achievement, more homework procrastination, less homework persistence, as well as lower academic self-concept, and lower expectancy to be able to master their homework (Trautwein et al., 2006; Dumont et al., 2012; Moroni et al., 2015; Guill et al., 2020).

To sum up, on the basis of the SDT, it can be theoretically argued that parental *responsiveness* and *structure* have the potential to positively influence students’ academic functioning by supporting their psychological needs and, thus, promoting their intrinsic motivation. High intrinsic motivation can be assumed to be favorable for (a) students’ academic achievement because it promotes high-quality learning (Ryan and Deci, 2020) and for (b) students’ homework behavior because it is more likely that students make great effort and procrastinate less if they are intrinsically motivated. In contrast, *control* is assumed to be detrimental to students’ academic functioning because it undermines students’ psychological needs and, thus, decreases their intrinsic motivation. Therefore, in the present study, we used responsiveness, structure, and control to measure the quality of parental homework assistance and we expected to find favorable relationships for responsiveness and structure and unfavorable relationships for control.

### 1.2. Reciprocal relationships between the quality of parental homework assistance and students’ academic functioning

Researchers have proposed that parents are likely to adapt their involvement to students’ competencies and their level of motivation and behavior (Grolnick and Slowiaczek, 1994; Grolnick and Apostoleris, 2002). Grolnick (2003) discussed different types of pressure related to parental involvement that can evoke controlling behavior in parents and, thus, can contribute to low-quality parental involvement. Among other sources, this pressure can come from contextual factors such as economic stress (referred to as *pressure from above*) or from a child’s low academic functioning (referred to as *pressure from below*; Grolnick, 2003; Dumont et al., 2014; Grolnick and Pomerantz, 2022). For example, parents may be alarmed about their child’s low academic achievement and, as a reaction to that, feel the need to exercise more control regarding their child’s homework (behavior). Along these lines, it has been argued that the relationship between parental involvement and students’ academic functioning is reciprocal (Grolnick and Slowiaczek, 1994; Bronstein et al., 2005), with students’ academic functioning being both the outcome and the antecedent of parental involvement.

Indeed, previous research at the secondary level has provided evidence for reciprocal links between the quality of parental homework assistance and students’ academic functioning: as described above, several studies found that the quality of parental homework assistance predicted children’s academic functioning (e.g., Dumont et al., 2014; Moroni et al., 2015; Guill et al., 2020). Studies that looked at the effects of students’ academic functioning on the quality of parental homework assistance or at reciprocal relationships found that low levels of academic functioning (i.e., low achievement, unfavorable homework behavior, low parents’ perceptions of their child’s efficacy) predicted a lower quality of homework assistance (i.e., more control and interference, lower cognitive engagement and autonomy support; Niggli et al., 2007; Dumont et al., 2014; Gonida and Cortina, 2014; Núñez et al., 2017; Xu et al., 2018). Whereas Grolnick’s (2003) theoretical assumptions of *pressure from below* relate to controlling behavior only, Dumont et al. (2014) found effects of students’ academic functioning on all three dimensions of parental homework assistance (i.e., low academic functioning was related to lower responsiveness and structure and higher control). In this context, it is noteworthy that Dumont et al. (2014) found that students’ homework behavior was linked to the positive forms of homework assistance (responsiveness and structure), whereas achievement was linked to parental control, pointing to the possibility that the individual quality dimensions might be linked differentially to different student outcomes.

In sum, both theoretical assumptions and empirical evidence point to bidirectional relationships between parental homework assistance and students’ academic functioning in secondary school. Because elementary school students have been studied less and previous results obtained for this population have been inconclusive, in the present study, we explicitly examined these reciprocal links in a sample of elementary school students.

### 1.3. Homework assistance for elementary school children

Extensive cross-sectional and longitudinal research has studied the relationship between parental homework assistance and students’ academic functioning in secondary school (e.g., Trautwein et al., 2006; Dumont et al., 2012, 2014; Moroni et al., 2015; Xu et al., 2018; Feng et al., 2019; Guill et al., 2020). However, as Nordt (2013) pointed out, findings on older students cannot simply be transferred to younger students. The reasons for this are that both students’ needs and their educational environment change over the course of different grade levels and with transitions to different school types. Both Eccles et al. (1993) and Hoover-Dempsey and Sandler (1995) highlighted the importance of the compatibility between students’ developmental stage and their educational environment (i.e., its characteristics and demands) for their educational outcomes. They proposed that students benefit most if their educational environment matches their developmental needs and provides opportunities for growth. Thus, although students’ needs as proposed by the SDT and the quality dimensions of homework assistance derived from it are generally valid regardless of the developmental stage, it is still possible that there are age- and grade-level differences in the relative importance of the dimensions and in how easily students’ needs can be satisfied. For example, it might be easier for parents to meet younger students’ needs because younger children usually have more positive attitudes towards both schoolwork, such as homework, and parental attention, such as help, praise, or interest (Hoover-Dempsey and Sandler, 1995). Moreover, the move from childhood to adolescence and the transition to secondary school increase opportunities for students to experience autonomy outside their home (e.g., by spending more time with their peers), which leads students to be more independent and to gain more autonomy from their parents (Eccles et al., 1993; Hoover-Dempsey and Sandler, 1995). In contrast to secondary school students, younger students might respond less negatively to parental control during homework assistance – as a type of behavior that undermines autonomy – because their need for autonomy is still less pronounced.

Regarding the quality dimensions that have been found to be relevant in secondary school samples (Dumont et al., 2014), prior studies in elementary school samples only found weak evidence for a link between parental *responsiveness* and students’ academic functioning (Núñez et al., 2015; Silinskas and Kikas, 2019). Whereas parental support (which is comparable with responsiveness) did not predict students’ achievement in any of the studies, Silinskas and Kikas (2019) found a positive link with students’ homework behavior (i.e., increased task persistence). Regarding *structure* at elementary school, to the best of our knowledge, only Cunha et al. (2018) included this dimension of parental homework assistance. They reported a negative correlation of parental environment-time management with students’ achievement. Because of the cross-sectional design of the study, the direction of the link is unclear; the negative association might also mirror parents’ increasing structure when their children are struggling with their academic achievement. However, bidirectional links between students’ academic functioning and the two positive forms of parental homework assistance (i.e., responsiveness and structure) have not yet been studied in elementary school. For parental *control* in elementary school, prior studies also only found weak evidence for links with students’ academic functioning. Only Núñez et al. (2015) reported a significant link with students’ achievement, whereas other studies did not report significant associations (Gonida and Cortina, 2014; Wu et al., 2022). Studies that looked into bidirectional effects provided first evidence that students’ academic functioning (i.e., parents’ perceptions of their child’s efficacy, students’ self-concept, students’ reading and math skills) might predict controlling and monitoring parental behavior in elementary school (Silinskas et al., 2013; Gonida and Cortina, 2014; Silinskas and Kikas, 2019). Taken together, with the exception of the associations between students’ prior academic functioning and subsequent controlling parental behavior, findings regarding possible reciprocal associations between the quality of parental homework assistance and students’ academic functioning in elementary school are inconclusive.

To sum up, although students’ needs as proposed by the SDT and the quality dimensions of parental homework assistance are generally valid regardless of students’ developmental stage, it is still possible that there are age-related differences in how easily students’ needs can be satisfied and in the relative importance of the quality dimensions. Because prior research in elementary school has been largely inconclusive, we examined whether there are reciprocal relationships between the quality of parental homework assistance and students’ academic functioning at the elementary school level that are similar to those that have been found at the secondary school level.

### 1.4. Homework assistance at school

In the past decades, homework assistance has partly shifted from the home to institutional environments. Following Sauerwein and Rother (2022), the increasing prevalence of institutional homework assistance has two major reasons. First, institutional homework assistance takes over part of the care work while parents are working in their jobs and thereby contributes to the compatibility of family and work life. Second, institutional homework assistance has the potential to decrease social inequalities by providing assistance to students who do not receive (high-quality) homework assistance at home (Nordt, 2020). In the context of the present study – elementary school students in Germany – homework assistance outside the students’ home is often provided as part of full-day school programs.^1^ In fact, 89% of all elementary full-day schools offer this type of support (StEG-Konsortium, 2019). Besides full-day schools, homework assistance is also provided by after-school care programs (e.g., *Horte* or *Betreute Grundschulen*), which take place in different settings and have different providers. As the settings are quite similar in all forms of assistance, we subsume them under the term “homework assistance at school” in the present study. Traditionally, German elementary schools were organized as half-day schools, with children spending their afternoon and, thus, their homework time at home. Although full-day programs have been extended in Germany, still only about half of all elementary school students participate in a full-day school program (e.g., in 2019 about 47%; the Secretariat of the Standing Conference of the Ministers of Education and Cultural Affairs in the Federal Republic of Germany, 2021). Homework assistance at school is often organized on a voluntary basis; it takes place in a group setting in the afternoon after regular classes are finished. It is usually provided by pedagogical staff and less often by teachers or university students (Brisson and Theis, 2020; Sauerwein and Rother, 2022). Nordt (2013) described the infrastructure at school, for instance, quiet rooms to work in and the provision of materials, as an advantage of homework assistance at school.

Because the SDT (Ryan and Deci, 2000) proposes universal psychological needs, it can be used across contexts to assess the quality of homework assistance. Therefore, the SDT (Ryan and Deci, 2000) also offers a useful theoretical framework for the assessment of scholastic homework assistance, which is similar to parental homework assistance. Moreover, qualitative empirical research has described quality dimensions that are related with the dimensions derived from the SDT (Huang and Cho, 2009; Nordt, 2013; Gordon et al., 2020). With regard to the quality dimension of *responsiveness*, descriptions of high-functioning homework assistance at school included comparable behavior such as listening to the children’s thoughts during homework preparation, creating positive and open environments, offering emotional and social support, and being responsive and empathetic (Huang and Cho, 2009; Nordt, 2013; Gordon et al., 2020). Regarding *structure*, prior studies reported that some homework assistants at school placed high value on setting rules that refer to the organization of the work space (e.g., students should have all important working materials with them; Nordt, 2013). They also reported that developing helpful study skills (e.g., time management, organizational skills) is one important aspect of homework assistance at school (Huang and Cho, 2009; Gordon et al., 2020). Nordt (2013) described *controlling* homework assistance at school as being characterized by many rules (e.g., students should be quiet and should not walk around), by punishments if a student does not follow the rules, by a strict and authoritarian appearance of the supervisor, or by critical feedback that is provided even if the student does not ask for help or for feedback. Moreover, the results obtained by Nordt (2013) provide first indications that the relationship between control at school and students’ academic functioning might be reciprocal because some supervisors stated that they applied particularly strict rules when students showed less adaptive behavior (e.g., when they did not follow rules or when they struggled with concentration). However, there is a lack of quantitative empirical research on the quality dimensions of homework assistance at school and on links to student outcomes.

Although the SDT (Ryan and Deci, 2000) can be applied to a variety of contexts, and qualitative research on scholastic homework assistance has proposed quality dimensions similar to those of parental homework assistance (Dumont et al., 2014), it is important to test whether the associations with students’ academic functioning in the school setting are similar to those in the parental setting. Given that, unlike parental homework assistance, homework assistance at school is provided by (at least partially) trained personnel who can be assumed to be familiar with the benefits of need-supportive behavior (Guill et al., 2020), the quality of homework assistance might be higher in the scholastic than in the parental context. However, because homework assistance at school often takes place in a group setting, whereas parental homework assistance is predominantly given in one-on-one settings, scholastic homework assistance might influence students’ achievement and homework behavior less than parental homework assistance. Regarding students’ academic functioning as an antecedent of the quality of homework assistance, these links might also be weaker in the scholastic than in the parental setting because homework assistants at school might feel less pressure if students have low academic achievement as they are more distanced from the students’ school careers.

In sum, homework assistance at school is highly important and becoming more and more common. Thus, empirical research on the quality of homework assistance at school and its links to students’ academic functioning is sorely needed. Therefore, in the present study, we applied the SDT (Ryan and Deci, 2000) to the context of homework assistance at school by examining possible reciprocal relationships between the quality dimensions proposed for parental homework assistance and students’ academic functioning.

### 1.5. The present study

The present study had two aims. The first aim was to examine whether there are reciprocal relationships between the quality of parental homework assistance and students’ academic functioning at the elementary school level (Research Question 1 [RQ1]). The second aim of our study was to exploratively investigate possible reciprocal relationships between the quality of homework assistance and students’ academic functioning for the context of scholastic homework assistance (Research Question 2 [RQ2]).

To measure students’ academic functioning, we followed prior research (Dumont et al., 2014; Guill et al., 2020) and included four indicators of students’ academic functioning. First, we used two indicators to measure academic achievement, namely, students’ grades and a test score. Although test results are better suited to reliably assess students’ achievement, students’ grades might more directly influence the homework process as parents might be better informed about students’ grades than about test results (Dumont et al., 2014). Whereas prior research focused on academic achievement in the reading domain (Dumont et al., 2014; Moroni et al., 2015), we assessed students’ grades in three subjects (mathematics, German, and social studies) and conducted a mathematics test. Second, we used two indicators of homework behavior, namely, homework effort and homework procrastination (Dumont et al., 2014). Students’ homework behavior is a relevant indicator of students’ academic functioning because it is one of the main goals of homework to improve students’ study skills and their self-regulated learning (Cooper and Valentine, 2001; Ramdass and Zimmerman, 2011; Dumont et al., 2014), which – in turn – might improve students’ academic achievement.

RQ1: On the basis of the SDT (Ryan and Deci, 2000) and its application in the context of homework assistance (Dumont et al., 2014), as well as prior research on secondary school students (e.g., Moroni et al., 2015; Guill et al., 2020), we expected parental *responsiveness* and *structure* to be positively and reciprocally related with students’ academic achievement and homework effort, while we expected them to be negatively associated with students’ procrastination (Hypothesis 1a). In contrast, we expected parental control to be negatively and reciprocally related with students’ academic achievement and homework effort, whereas we expected it to be positively and reciprocally related with homework procrastination (Hypothesis 1b).

RQ2: To explore possible links between the quality of homework assistance at school and students’ academic functioning, we used a subsample of students who received scholastic homework assistance in addition to parental homework assistance. In line with our hypotheses regarding parental homework assistance, we expected scholastic responsiveness and structure to be positively and reciprocally related with students’ academic achievement and homework effort, while we expected them to be negatively associated with students’ procrastination (Hypothesis 2a). Further, we expected scholastic control to be negatively and reciprocally related with students’ academic achievement and homework effort, whereas we expected it to be positively and reciprocally related with homework procrastination (Hypothesis 2b).

The present study adds to previous research in two ways: First, we focused on elementary school students, a population under-researched in previous studies. Second, we explored associations in the context of homework assistance at school, a context of children’s schooling that is growing in importance.

## 2. Materials and methods

### 2.1. Sample

We drew on longitudinal data from two points of measurement that were collected for the purpose of this study. In June 2019, after the study was approved by the responsible ministry (Ministry of Education, Science, Research, and Culture in Schleswig-Holstein), 100 elementary schools (half-day and full-day schools of all types) were contacted, of which 10 schools agreed to participate in the study. The first point of measurement was from September to October 2019 (T1); the second was from February to March 2020 (T2) and ended before schools had to close due to the COVID-19 pandemic. Participation in the study was voluntary; written consent was obtained from the parents. Students did not have any disadvantages if they decided not to participate in the study.

To test Hypotheses 1a and 1b (parental homework assistance), we drew on data from 335 fourth graders (50.2% girls) in 21 classes who participated in at least one of the two points of measurement (T1: *n* = 312, T2: *n* = 277). In Schleswig-Holstein, Grade 4 is the last grade of elementary school and students are assigned to different secondary school tracks based on their academic achievement at the end of elementary school. Thus, the students in our sample were relatively heterogeneous regarding their performance. In the total sample, 23.6% of the students stated that they spoke another language besides German at home, which indicates a migration background. Regarding the education of their parents, 55.6% of the students stated that at least one of their parents had the qualification to go to university (i.e., had obtained the school leaving certificate *Abitur*), while 33.5% did not know whether their parents had this qualification.

To test Hypotheses 2a and 2b (homework assistance at school), we used data from a subsample of 112 students (57.3% girls) who additionally provided information on scholastic homework assistance on at least one of the two points of measurement (T1: *n* = 85, T2: *n* = 75). In the subsample, 20.7% indicated that they had a migration background and 49.5% stated that their parents had Abitur, while 37.1% did not know whether their parents had this qualification.

### 2.2. Instruments

All constructs were measured at both time points using the same instruments (the items are displayed in the Supplementary material in Supplementary Table S1). The data collection took place during regular school hours in class and lasted one school period (45 min). Each student first completed a paper-pencil mathematics test that took 12 min and afterwards filled in an online questionnaire on a tablet handed out by the administrators. The data collection was administered by trained research assistants who were undergraduate psychology students at the time.

#### 2.2.1. Quality of homework assistance

Three dimensions of homework quality – responsiveness, structure, and control – were assessed using items from Dumont et al. (2014). Students rated the quality of the homework assistance they received on a 4-point Likert scale (1 = *completely disagree* to 4 = *completely agree*). Originally, the items referred to parental assistance only, but they were adapted in the present study to assess scholastic assistance as well. That is, the items were mostly used in parallel form, with “supervisor” replacing “parent” for the items referring to scholastic homework assistance. Students from the subsample that received any form of scholastic homework assistance answered the items twice, once for the homework setting at home (14 items) and once for the homework setting at school (12 items because two items measuring structure were not applicable for homework assistance at school). Responsiveness was measured with four items (Cronbach’s α/McDonald’s ω: parental: α_T1_ = 0.55/ω_T1_ = 0.57, α_T2_ = 0.73/ω_T2_ = 0.73; scholastic: α_T1_ = 0.66/ω_T1_ = 0.69, α_T2_ = 0.72/ω_T2_ = 0.74). Structure was operationalized with six items for parental homework assistance and with four items for scholastic homework assistance (parental: α_T1_ = 0.67/ω_T1_ = 0.67, α_T2_ = 0.69/ω_T2_ = 0.69; scholastic: α_T1_ = 0.65/ω_T1_ = 0.63, α_T2_ = 0.70/ω_T2_ = 0.71). Control was assessed with four items (parental: α_T1_ = 0.62/ω_T1_ = 0.64, α_T2_ = 0.67/ω_T2_ = 0.67; scholastic: α_T1_ = 0.84/ω_T1_ = 0.84, α_T2_ = 0.85/ω_T2_ = 0.85).

#### 2.2.2. Academic functioning

We used four indicators to measure students’ academic functioning: (a) report card grades, (b) a mathematics test, (c) homework effort, and (d) homework procrastination. Report card grades and the mathematics test are indicators of academic achievement, whereas homework effort and homework procrastination are indicators of homework behavior.

##### 2.2.2.1. Academic achievement

Students stated their last report card grades in mathematics, German, and social studies on a scale ranging from one (highest grade) to six (lowest grade). In the German school system, teachers have the option of adding a plus or a minus to grades (for example, 2+ for a student who achieves slightly better than a 2). We used the average of the grades in the three subjects and recoded the grades, including plus and minus, so that high values indicated better grades (1 = *lowest grade* to 13 = *highest grade*).^2^ Moreover, students completed a mathematics test that was adapted from Lipowsky et al. (2011), which captured the four basic arithmetic operations (addition, subtraction, multiplication, and division). The test consisted of 28 items at T1 and 31 items at T2, which were distributed across six pages with three to eight items on each page (α_T1_ = 0.85, α_T2_ = 0.88). Students had limited time for each page and were not allowed to go back to a previous page when the time was up (1.5 to 2 min per page). The items were scored as right (= *1 point*) or wrong (= *0 points*) and the score of each item was added up to a total score (0 = *lowest score* to 28 [T1]/31 [T2] = *highest score*).

##### 2.2.2.2. Homework behavior

Students rated their homework behavior regarding effort and procrastination on a 4-point Likert scale (1 = *completely disagree* to 4 = *completely agree*; the items are displayed in the Supplementary material, Supplementary Table S1). Homework effort was assessed using five items that were adapted from Trautwein et al. (2006; α_T1_ = 0.81/ω_T1_ = 0.81, α_T2_ = 0.84/ω_T2_ = 0.85). One item for homework effort, which captured the amount of homework that students did as well as they could, was measured on a different scale (1 = *none* to 4 = *all*). Procrastination was measured with three items (Dumont et al., 2014; α_T1_ = 0.70/ω_T1_ = 0.71, α_T2_ = 0.78/ω_T2_ = 0.79).

#### 2.2.3. Covariates

Students also reported on their gender (1 = *male*, 2 = *female*) and on their family background in terms of a possible migration background and parental education. As an indicator of migration background, students were asked whether they spoke a language other than German at home (1 = *yes*, 2 = *no*). To operationalize parents’ educational background, students stated whether their parents had a qualification for university study (in Germany: Abitur; 1 = *yes*, 2 = *no*, 3 = *I do not know*). We coded this information into two dummies; the first one indicated whether parents had Abitur (0 = *no*/*I do not know*, 1 = *yes*), the second one indicated whether students knew whether their parents had Abitur (0 = *yes*/*no*, 1 = *I do not know*). To make full use of the information from both points of measurement, we combined information from T1 and T2 for each covariate.

### 2.3. Statistical analyses

To get an overview of the data, we first computed descriptive statistics (reliabilities, means, standard deviations, skewness, and kurtosis) and bivariate correlations in SPSS (Version 27).The main analyses were calculated in M*plus* 8.5 (Muthén and Muthén, 1998–2017). We used structural equation modeling (SEM; e.g., Little, 2013) to study the reciprocal relationships between parental homework assistance and students’ academic functioning. Parental responsiveness, structure, and control, as well as students’ effort and procrastination, were specified as latent variables, while students’ grades and test results were modeled as manifest variables. Prior to the main analysis, we tested measurement invariance across time by using confirmatory factor analysis. Therefore, we tested configural, metric, and scalar invariance for each latent variable. The fit indices of each level of measurement invariance are displayed in Table 1. To compare the models, we evaluated differences in CFI (ΔCFI) by comparing the configural with the metric and scalar invariance; values greater than or equal to −0.01 indicated equivalent model fit (Cheung and Rensvold, 2002). The ΔCFI ranged between −0.02 and 0.00 (configural vs. metric) and − 0.04 and 0.00 (configural vs. scalar). Although the ΔCFI exceeded the cut-off value proposed by Cheung and Rensvold (2002) for students’ procrastination and parental control, for reasons of parsimony (i.e., limiting the number of parameters that needed to be estimated in SEM), the factor loadings were modeled as invariant across time in all models in the following steps of the analyses. Moreover, the error terms of each indicator were allowed to correlate across time.

**Table 1:** Measurement invariance of the quality dimensions of parental homework assistance and homework behavior across time.

Variable	Fit indices						Model comparison
	*χ* ^2^	df	CFI	TLI	RMSEA	SRMR	ΔCFI
*Responsiveness*							
Configural	32.85	15	0.96	0.93	0.06	0.05	
Metric	39.60	18	0.95	0.93	0.06	0.06	–0.01^a^
Scalar	43.05	21	0.95	0.93	0.06	0.07	–0.01^b^
*Structure*							
Configural	68.57	47	0.96	0.94	0.04	0.05	
Metric	72.38	52	0.96	0.95	0.04	0.06	0.00^a^
Scalar	80.73	57	0.95	0.95	0.04	0.06	–0.01^b^
*Control*							
Configural	34.66	15	0.95	0.90	0.07	0.04	
Metric	42.48	18	0.93	0.90	0.07	0.05	–0.02^a^
Scalar	53.48	21	0.91	0.88	0.07	0.05	–0.04^b^
*Effort*							
Configural	37.97	29	0.99	0.99	0.03	0.03	
Metric	41.03	33	0.99	0.99	0.03	0.04	0.00^a^
Scalar	45.12	37	0.99	0.99	0.03	0.05	0.00^b^
*Procrastination*							
Configural	18.45	5	0.96	0.89	0.09	0.04	
Metric	28.35	7	0.94	0.88	0.10	0.06	–0.02^a^
Scalar	30.27	9	0.94	0.91	0.09	0.05	–0.02^b^

*Note.* CFI = comparative fit index; TLI = Tucker-Lewis index; RMSEA = root mean square error of approximation; SRMR = standardized root mean square residual.

a Comparing configural with metric invariance.

b Comparing configural with scalar invariance.

To study the relationship between scholastic homework assistance and students’ academic functioning, we used a different approach because the sample size was relatively small (*n* = 112 students participated in at least one point of measurement). In SEM, high model complexity in combination with a small sample size can lead to estimation problems (Ulitzsch et al., 2021). To decrease model complexity, we used a single-indicator approach (Hayduk and Littvay, 2012; Savalei, 2019). In the single-indicator approach, the latent variable is measured with a composite indicator (i.e., scale score), and the error variance is fixed to the measurement error variance. More specifically, for scholastic responsiveness, structure, and control and for students’ effort and procrastination, we set the loading of the composite indicator to one and fixed the measurement error variance to *s*2(1 − rel), where *s*2 is the observed variance of the indicator and rel is an estimate of the composite’s score reliability (Cronbach’s alpha).

**Figure 1:**
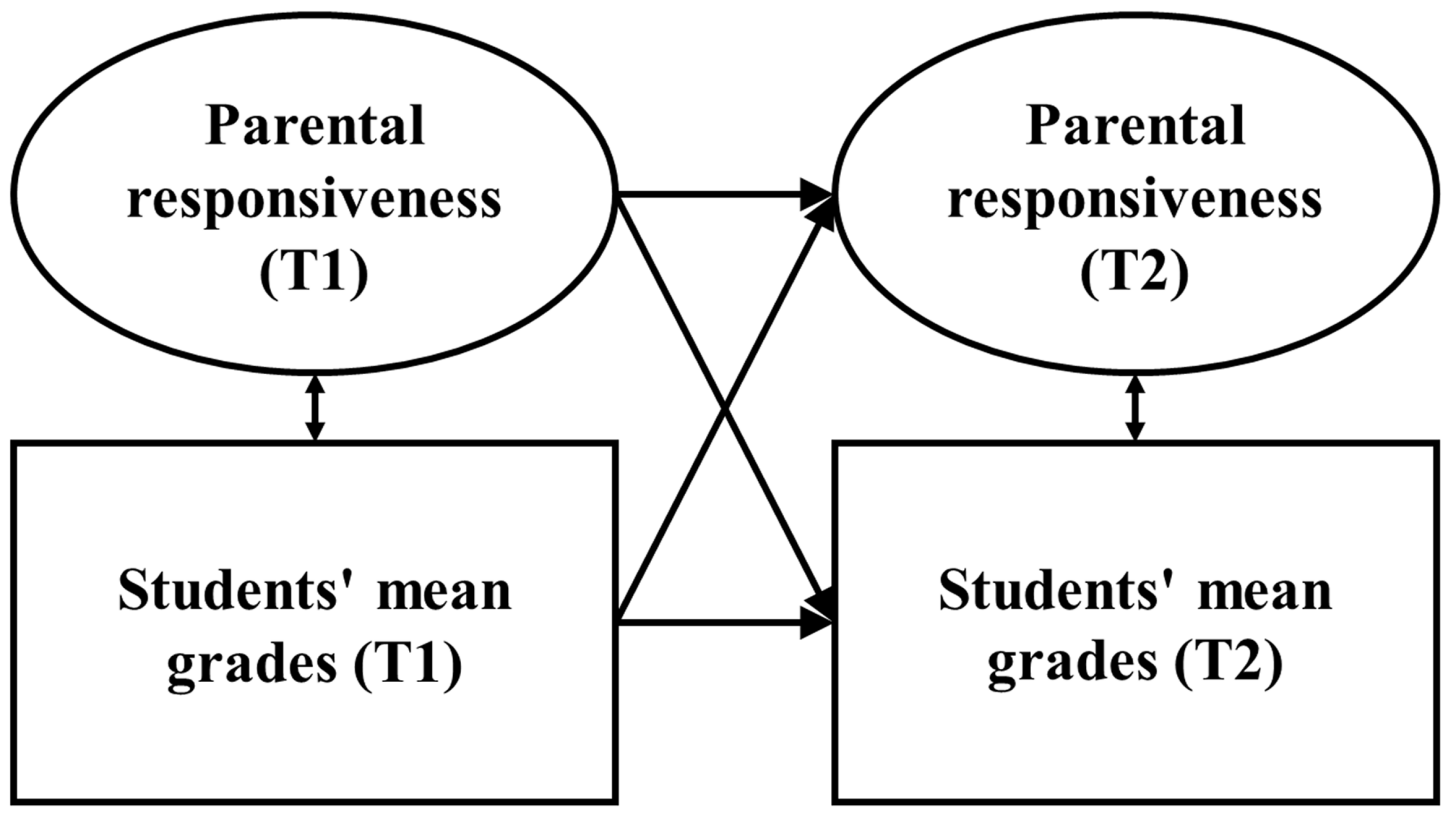
Analytical model of the reciprocal relationship between parental responsiveness and students’ mean grades. Parental responsiveness and students’ mean grades at T2 were regressed on the covariates. T1/T2 = first/second point of measurement.

For the main analysis, we calculated cross-lagged panel models with homework assistance quality at T1 predicting academic functioning at T2 and vice versa, controlling for student gender, migration background, and parental education (i.e., homework assistance quality and academic functioning at T1 and T2 were regressed on the covariates). The models were calculated separately for each quality dimension of parental and scholastic homework assistance (responsiveness, structure, and control) and each indicator of students’ academic functioning (mean grade, test result, effort, and procrastination). Thus, each model included only one quality dimension and only one student outcome, resulting in 12 models for parental homework assistance and 12 models for scholastic homework assistance. Figure 1 illustrates how these models were constructed using the example of parental responsiveness as a quality dimension and students’ mean grades as an indicator of students’ academic functioning.

### 2.4. Missing data

The amount of missing data ranged between 7 and 32% in the total sample. Within the group of students who received scholastic homework assistance, the amount of missing data on the variables on the quality of scholastic homework assistance ranged between 26 and 37%. To handle missing data, full information maximum likelihood estimation, integrated into M*plus*, was used (Enders, 2010).

## 3. Results

### 3.1. Descriptives

The means, standard deviations, and bivariate intercorrelations are displayed in Table 2 (intercorrelations at T1), Table 3 (intercorrelations at T2), and Table 4 (intercorrelations between T1 and T2). Students reported relatively high (i.e., higher than the midpoint of the scale) mean parental responsiveness and structure as well as relatively low control at both points of measurement. To compare the quality of parental and scholastic homework assistance, we conducted paired *t*-tests for each quality dimension (e.g., parental responsiveness at T1 vs. scholastic responsiveness at T1) in the subsample of students who had provided information on both parental and scholastic homework assistance. Students reported levels of scholastic responsiveness and control that were similar to those of parental homework assistance at both points of measurement (T1/T2: *p* > 0.05), but they reported significantly lower levels of scholastic structure than parental structure at both points of measurement (T1: *t*[79] = 5.99, *p* < 0.001; T2: *t*[72] = 7.00, *p* < 0.001).

**Table 2:** Intercorrelations between the manifest variables at T1.

	*M*/%	*SD*	Skewness	Kurtosis	1	2	3	4	5	6	7	8	9	10	11	12
1. Mean grades (T1)	9.42	2.10	−0.61	0.56												
2. Test score (T1)	17.41	4.43	−0.33	0.16	**0.29**											
3. Effort (T1)	3.38	0.55	−1.47	3.29	**0.17**	0.09										
4. Procrastination (T1)	1.85	0.78	0.81	−0.07	**−0.25**	**−0.13**	**−0.48**									
5. Parental responsiveness (T1)	3.39	0.53	−0.93	0.67	−0.02	0.12	**0.15**	0.00								
6. Parental structure (T1)	3.32	0.54	−0.85	0.13	0.03	0.04	**0.20**	−0.03	**0.30**							
7. Parental control (T1)	1.71	0.65	0.97	0.24	**−0.20**	−0.02	**−0.18**	**0.27**	**0.16**	0.05						
8. Scholastic responsiveness (T1)	3.38	0.62	−1.29	2.14	0.08	0.18	0.05	−0.06	**0.36**	**0.39**	−0.02					
9. Scholastic structure (T1)	2.87	0.73	−0.32	−0.58	−0.22	−0.06	0.09	−0.02	0.07	**0.28**	0.00	**0.29**				
10. Scholastic control (T1)	1.62	0.76	1.54	1.95	**−0.40**	−0.06	−0.12	**0.40**	−0.01	0.02	**0.48**	0.08	0.19			
11. Gender^a^	50.20%	**–**	**–**	**–**	0.08	**−0.14**	**0.17**	−0.05	0.09	0.08	−0.06	0.10	−0.05	**−0.31**		
12. Migration background^b^	23.60%	**–**	**–**	**–**	0.01	−0.05	0.01	0.05	0.10	0.00	−0.09	0.08	−0.12	**−0.25**	**0.13**	
13. Qualification for university study^c^	55.60%	**–**	**–**	**–**	**0.22**	**0.25**	−0.06	−0.02	0.07	**0.17**	0.03	**0.23**	0.13	0.10	−0.03	**−0.21**

*Note.* T1 = first point of measurement. Statistically significant correlations at *p* ≤ 0.05 are in bold. Reference categories are given below.

a Male.

b Migration background.

c No qualification for university/I do not know.

**Table 3:** Intercorrelations between the manifest variables at T2.

	*M*/%	*SD*	Skewness	Kurtosis	1	2	3	4	5	6	7	8	9	10	11	12
1. Mean grades (T2)	9.35	2.39	**−**0.69	0.52												
2. Test score (T2)	16.91	5.59	0.18	−0.58	**0.35**											
3. Effort (T2)	3.38	0.56	−1.20	1.74	**0.34**	0.05										
4. Procrastination (T2)	1.77	0.80	1.06	0.37	**−0.39**	**−0.24**	**−0.43**									
5. Parental responsiveness (T2)	3.46	0.59	−1.61	2.54	0.00	−0.04	**0.20**	**−0.19**								
6. Parental structure (T2)	3.38	0.51	−0.80	0.24	0.03	−0.08	**0.23**	−0.10	**0.36**							
7. Parental control (T2)	1.61	0.66	1.41	1.96	**−0.29**	**−0.20**	−0.12	**0.28**	−0.02	−0.02						
8. Scholastic responsiveness (T2)	3.41	0.56	−1.69	4.13	0.15	−0.10	**0.42**	**−0.26**	0.12	0.12	−0.14					
9. Scholastic structure (T2)	2.94	0.70	−0.42	−0.27	0.01	−0.06	**0.42**	−0.09	0.06	**0.37**	0.01	**0.57**				
10. Scholastic control (T2)	1.80	0.89	1.03	0.12	−0.10	−0.12	0.06	**0.27**	−0.10	0.06	**0.45**	−0.02	**0.33**			
11. Gender^a^	50.20%	**–**	**–**	**–**	0.09	**−0.15**	**0.16**	−0.02	**0.14**	0.05	**−0.19**	0.15	0.20	−0.08		
12. Migration background^b^	23.60%	**–**	**–**	**–**	0.05	−0.11	0.06	−0.02	**0.23**	−0.04	**−0.19**	−0.12	**−0.31**	**−0.30**	**0.13**	
13. Qualification for university study^c^	55.60%	**–**	**–**	**–**	0.13	**0.15**	**−0.14**	0.03	0.02	0.09	0.03	−0.22	−0.09	0.07	−0.03	**−0.21**

*Note.* T2 = second point of measurement. Statistically significant correlations at *p* ≤ 0.05 are in bold. Reference categories are given below.

a Male.

b Migration background.

c No qualification for university/I do not know.

**Table 4:** Intercorrelations between the manifest variables at T1 (table rows) and T2 (table columns).

	T2									
	1	2	3	4	5	6	7	8	9	10
1. Mean grades (T1)	**0.60**	**0.31**	**0.16**	**−0.37**	0.05	0.04	**−0.24**	**0.26**	−0.01	−0.12
2. Test score (T1)	**0.24**	**0.59**	0.06	**−0.20**	0.05	−0.02	−0.05	−0.07	−0.01	−0.16
3. Effort (T1)	**0.33**	0.03	**0.56**	**−0.26**	0.12	**0.15**	−0.07	**0.26**	**0.31**	0.02
4. Procrastination (T1)	**−0.32**	−0.06	**−0.25**	**0.36**	**−0.16**	**−0.14**	−0.01	0.05	−0.03	0.13
5. Parental responsiveness (T1)	0.00	−0.05	0.02	0.02	**0.37**	**0.19**	0.01	−0.18	−0.18	−0.12
6. Parental structure (T1)	0.01	0.06	0.11	−0.01	0.12	**0.46**	**−0.15**	0.01	0.07	−0.06
7. Parental control (T1)	**−0.28**	−0.03	−0.04	**0.15**	0.09	0.07	**0.40**	−0.13	−0.05	**0.25**
8. Scholastic responsiveness (T1)	0.18	0.18	−0.02	−0.08	0.12	0.03	−0.09	0.19	0.07	−0.13
9. Scholastic structure (T1)	−0.23	−0.08	−0.11	−0.07	0.13	**0.34**	0.06	−0.10	**0.46**	**0.38**
10. Scholastic control (T1)	−0.23	−0.01	−0.05	**0.45**	−0.15	0.03	**0.44**	−0.06	0.01	**0.44**

*Note.* T1/T2 = first/second point of measurement. Statistically significant correlations at *p* ≤ 0.05 are in bold.

The quality dimensions were only partly associated with each other. One finding that was consistent across the environments and points of measurement was that responsiveness and structure were positively related with each other (0.29 ≤ *r* ≤ 0.57). In contrast, control showed different relationships depending on the environment and the point of measurement. While parental control was positively related with responsiveness at T1 (*r* = 0.16), scholastic control was positively related with structure at T2 (*r* = 0.33).

Of the three quality dimensions at T1, only control was significantly correlated with academic functioning at T2. That is, both parental and scholastic control at T1 were linked to more procrastination at T2 (parental: *r* = 0.15; scholastic: *r* = 0.45). Additionally, parental control was negatively linked to the mean grade (*r* = −0.28). The four indicators of academic functioning at T1 were only partly correlated with the quality dimensions of parental and scholastic homework assistance at T2, and the relationships varied between the two contexts. The only finding that was consistent across the two contexts was that effort at T1 and structure at T2 were positively correlated (parental: *r* = 0.15; scholastic: *r* = 0.31).

### 3.2. Cross-lagged models

In the following, we present our findings on the cross-lagged relationships between the three quality dimensions of parental and scholastic homework assistance (responsiveness, structure, and control) and the four indicators of students’ academic functioning (achievement: mean grade and test result; homework behavior: homework effort and homework procrastination). The sequence in which the findings are described follows the hypotheses as stated above.

#### 3.2.1. Parental homework assistance and academic functioning

**Figure 2:**
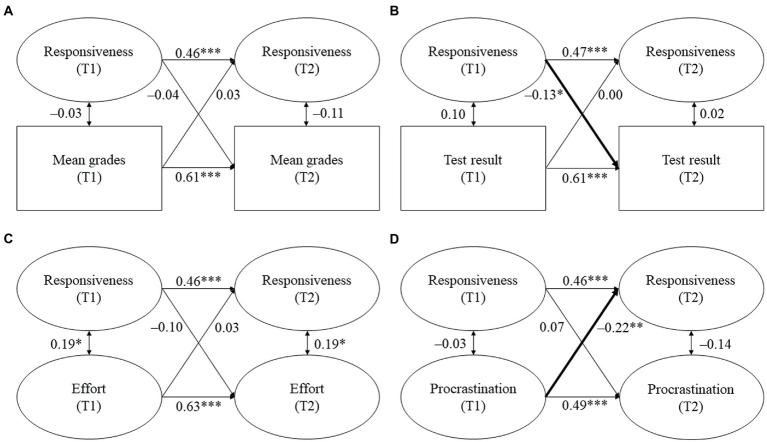
**(A–D)** Cross-lagged models for parental responsiveness and students’ academic functioning. Standardized coefficients. All covariates were included. Statistically significant cross-lagged coefficients are in bold. T1/T2 = first/second point of measurement. *p ≤ 0.05, **p ≤ 0.01, ***p ≤ 0.001.

**Figure 3:**
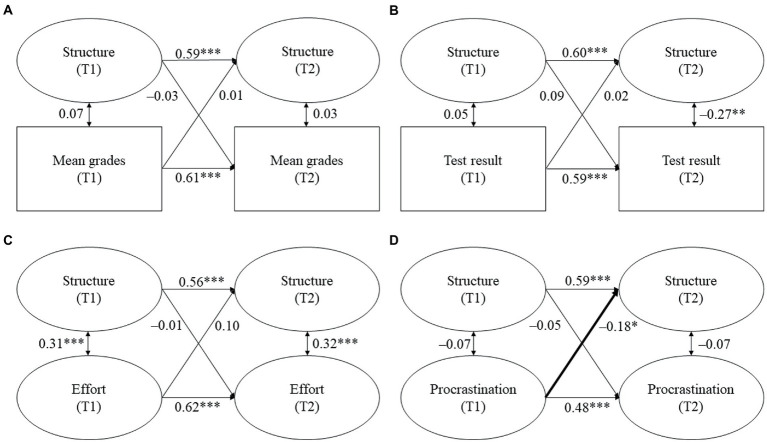
**(A–D)** Cross-lagged models for parental structure and students’ academic functioning. Standardized coefficients. All covariates were included. Statistically significant cross-lagged coefficients are in bold. T1/T2 = first/second point of measurement. *p ≤ 0.05, **p ≤ 0.01, ***p ≤ 0.001.

**Figure 4:**
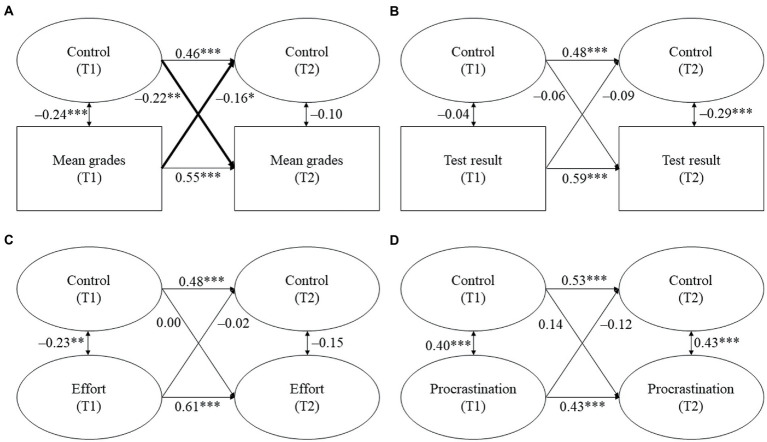
**(A–D)** Cross-lagged models for parental control and students’ academic functioning. Standardized coefficients. All covariates were included. Statistically significant cross-lagged coefficients are in bold. T1/T2 = first/second point of measurement. *p ≤ 0.05, **p ≤ 0.01, ***p ≤ 0.001.

The models for parental homework assistance are displayed in Figures 2–4. Tables displaying the detailed results can be found in the online Supplementary material. We report standardized coefficients (stdyx for all latent and manifest continuous outcomes and predictors and stdy for categorical variables, i.e., the covariates).

*Hypothesis 1a*: We expected parental responsiveness and structure to be positively and reciprocally related with students’ (a) mean grades, (b) test result, and (c) homework effort, while we expected them to be negatively associated with (d) homework procrastination.

Against our expectations, no significant links were found between perceived parental responsiveness and structure and students’ mean grades. Contrary to our hypothesis, parental responsiveness at T1 negatively predicted students’ test result at T2 (β = −0.13, *p* = 0.046, see Figure 2B). Thus, students scored lower on the mathematics achievement test at T2 the more parental responsiveness they perceived at T1. However, this relationship was not reciprocal because parental responsiveness at T2 was not predicted by students’ test result at T1. Moreover, parental structure was neither unidirectionally nor reciprocally related with students’ test result. In contrast to our hypothesis, parental responsiveness and structure were not related with students’ homework effort. However, while parental responsiveness and structure at T1 also did not predict students’ procrastination at T2, both responsiveness and structure at T2 were predicted by students’ procrastination at T1. Thus, students reported lower parental responsiveness (β = −0.22, *p* = 0.006, see Figure 2D) and parental structure at T2 (β = −0.18, *p* = 0.027, see Figure 3D) the more they procrastinated at T1.

To sum up, Hypothesis 1a was only minimally supported. Out of the expected links between parental responsiveness and structure and students’ academic functioning, only two proved to be significant: students’ prior procrastination predicted subsequent parental responsiveness and structure during homework assistance. However, we also found one relationship that was contrary to our expectations because prior parental responsiveness negatively predicted subsequent test results.

*Hypothesis 1b*: We expected parental control to be negatively and reciprocally related with students’ (a) mean grades, (b) test result, and (c) homework effort, whereas we expected it to be positively and reciprocally related with (d) homework procrastination.

In line with our hypothesis, we found a reciprocal association between parental control and students’ mean grades (see Figure 4A). Control at T1 negatively predicted students’ mean grades at T2 (β = −0.22, *p* = 0.002), while students’ mean grades at T1 negatively predicted control at T2 (β = −0.16, *p* = 0.041). Thus, students had lower subsequent mean grades the more they reported parental control during homework assistance. Vice versa, students perceived more subsequent control the lower their prior grades were. However, parental control was not associated with students’ test results or their homework behavior.

To sum up, Hypothesis 1b was partially supported because we found a reciprocal relationship between parental control and students’ mean grades, but we did not find a reciprocal relationship for the other indicators of academic achievement.

#### 3.2.2. Scholastic homework assistance and academic functioning

**Figure 5:**
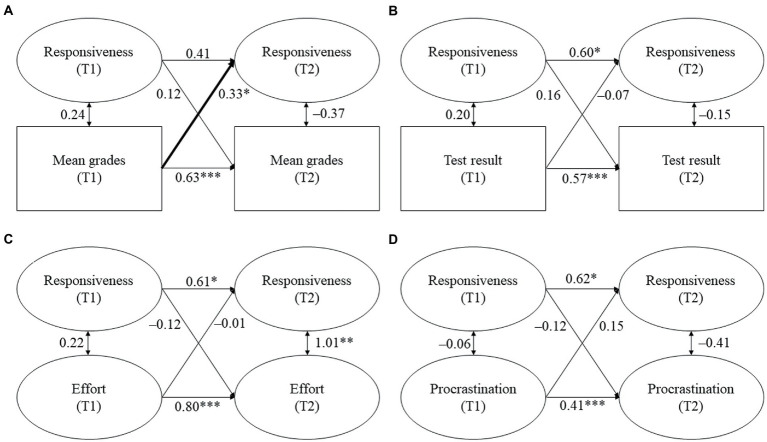
**(A–D)** Cross-lagged models for scholastic responsiveness and students’ academic functioning. Standardized coefficients. All covariates were included. Statistically significant cross-lagged coefficients are in bold. T1/T2 = first/second point of measurement. *p ≤ 0.05, **p ≤ 0.01, ***p ≤ 0.001.

**Figure 6:**
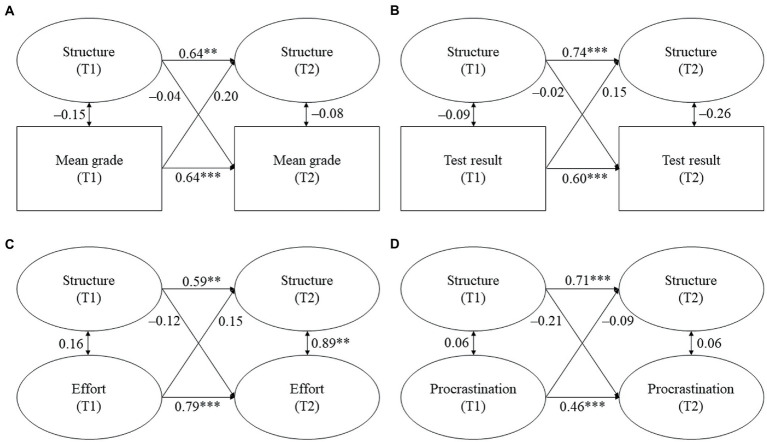
**(A–D)** Cross-lagged models for scholastic structure and students’ academic functioning. Standardized coefficients. All covariates were included. Statistically significant cross-lagged coefficients are in bold. T1/T2 = first/second point of measurement. *p = 0.05, **p ≤ 0.01, ***p ≤ 0.001.

**Figure 7:**
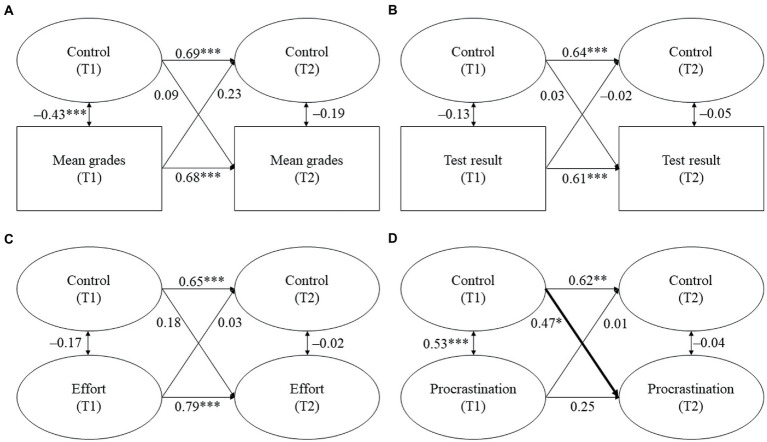
(A–D) Cross-lagged models for scholastic control and students’ academic functioning. Standardized coefficients. All covariates were included. Statistically significant cross-lagged coefficients are in bold. T1/T2 = first/second point of measurement. *p ≤ 0.05, **p ≤ 0.01, ***p ≤ 0.001.

The models for scholastic homework assistance are displayed in Figures 5–7. Tables displaying the results can be found in the online Supplementary material.

*Hypothesis 2a*: We expected scholastic responsiveness and structure to be positively and reciprocally related with students’ (a) mean grades, (b) test result, and (c) homework effort, while we expected them to be negatively associated with (d) students’ procrastination.

While responsiveness and structure during homework assistance at school at T1 did not predict students’ mean grades at T2, students’ mean grades at T1 positively predicted responsiveness at T2 (β = 0.33, *p* = 0.027, see Figure 5A) but did not predict structure. Therefore, students with higher prior mean grades perceived higher subsequent responsiveness during homework assistance at school. However, no significant relationships were found between scholastic responsiveness or structure and students’ test result or homework behavior.

To sum up, Hypothesis 2a was only minimally supported. We did not find that responsiveness or structure during homework assistance at school predicted any of the indicators of students’ academic functioning. However, students’ mean grades predicted scholastic responsiveness.

*Hypothesis 2b*: We expected scholastic control to be negatively and reciprocally related with students’ (a) mean grade, (b) test result, and (c) homework effort, whereas we expected it to be positively and reciprocally related with (d) homework procrastination.

No significant unidirectional or reciprocal relationships were found between control during homework assistance at school and students’ academic achievement or their homework effort. However, in line with our hypothesis, control during homework assistance at school at T1 positively predicted students’ homework procrastination at T2 (β = 0.47, *p* = 0.017, see Figure 7D). Therefore, students reported higher subsequent homework procrastination the more they perceived control during homework assistance at school. Vice versa, students’ homework procrastination at T1 did not significantly predict control during homework assistance at school at T2.

To sum up, Hypothesis 2b was only minimally supported because control predicted students’ homework procrastination but did not predict any of the other indicators of students’ academic functioning. Vice versa, students’ academic functioning did not predict control during homework assistance at school.

## 4. Discussion

Our study had two aims. The first aim was to examine reciprocal relationships between the quality of parental homework assistance and students’ academic functioning at the elementary school level, where previous findings have been inconsistent (e.g., Silinskas et al., 2013; Núñez et al., 2015; Cunha et al., 2018). The second aim of our study was to investigate whether there are reciprocal relationships between the quality of scholastic homework assistance and students’ academic functioning. Our reason for investigating this is the growing importance of scholastic homework assistance and the lack of quantitative research that addresses homework assistance in this environment.

The main findings are summarized in Table 5. Overall, we found a complex pattern of results. Regarding parental homework assistance, we found reciprocal relationships between parental control and mean grades. In addition, we found several unidirectional links, namely, a negative link between procrastination at T1 and both parental responsiveness and structure at T2. Unexpectedly, parental responsiveness at T1 was negatively related with students’ test score in a mathematics achievement test at T2. All other links did not reach statistical significance. Regarding scholastic homework assistance, only two unidirectional links proved to be significant: scholastic control at T1 was positively related with procrastination at T2, and mean grades at T1 positively predicted scholastic responsiveness at T2. There were no other statistically significant unidirectional or bidirectional links. In sum, even though several of the assumed associations were not found in the present study, the findings provide valuable information on the quality of homework assistance both at home and at school.

**Table 5:** Overview of the main findings.

Hypothesis	Quality dimension	Academic functioning	Parental		Scholastic	
			HA_T1_ ➔ AF_T2_	AF_T1_ ➔ HA_T2_	HA_T1_ ➔ AF_T2_	AF_T1_ ➔ HA_T2_
1a/2a	Responsiveness	Mean grades	o	o	o	0.33*
		Test score	−0.13*	o	o	o
		Effort	o	o	o	o
		Procrastination	o	−0.22**	o	o
	Structure	Mean grades	o	o	o	o
		Test score	o	o	o	o
		Effort	o	o	o	o
		Procrastination	o	−0.18*	o	o
1b/2b	Control	Mean grades	−0.22**	−0.16*	o	o
		Test score	o	o	o	o
		Effort	o	o	o	o
		Procrastination	o	o	0.47*	o

*Note.* o = significant link; T1/T2 = first/second point of measurement; AF = academic functioning; HA = homework assistance. *p ≤ 0.05; **p ≤ 0.01.

### 4.1. Parental homework assistance

#### 4.1.1. Links between parental responsiveness, structure, and students’ academic functioning (H1a)

Although previous studies reported that parental responsiveness and structure positively predicted students’ academic functioning at secondary school (e.g., Dumont et al., 2014; Moroni et al., 2015; Guill et al., 2020), the missing link in our study is in line with the findings of Núñez et al. (2015), who did not find significant relationships between parental support and academic functioning at elementary school. Similar to the present study, students in the study of Núñez et al. (2015) were at the end of elementary school – albeit in Grades 5 and 6 as elementary school in Spain comprises Grades 1 to 6. Therefore, the lack of significant relationships found might partly be due to the timing of Núñez et al.’s (2015) and our study. It is possible that the dynamics between students and their parents are relatively consolidated at the end of elementary school, which might make it harder to find changes in the quality of homework assistance and students’ academic functioning. This assumption is reflected in our study as we found relatively high stabilities of both the quality dimensions and the indicators of academic functioning (see Figures 2, 3). Moreover, parents of elementary school students might generally provide high levels of responsiveness and structure because their children’s study skills and self-management are not yet fully developed (Dufresne and Kobasigawa, 1989; Patall et al., 2008). At the same time, there might be less variation in younger students’ homework behavior because their intrinsic motivation is usually higher as intrinsic motivation only starts to decline with increasing age (Gottfried et al., 2001; Gnambs and Hanfstingl, 2016). Both assumptions are also reflected in our findings, as students perceived high mean responsiveness and structure and reported high mean effort and low mean procrastination (see Tables 2, 3). Taken together, high levels of parental responsiveness and structure, in combination with favorable homework behavior of the students, might be the reason for why we did not find significant relationships between the constructs.

The negative association we found between parental responsiveness and students’ mathematics test result is unexpected and incongruent with prior longitudinal research on parental support (Moroni et al., 2015). However, one other study (Cooper and Nye, 2000) also found a negative link between responsiveness, measured as the frequency of direct homework involvement (i.e., helping if it is needed), and students’ standardized test result. However, the finding of Cooper et al. was based on cross-sectional data and, therefore, might rather mirror increases in parental involvement if students have low academic achievement. One possible explanation for the negative link found in our study is that high levels of responsiveness might lead to students relying strongly on the help of their parents, which might make it difficult for students to take an achievement test on their own. However, this finding should be interpreted with caution because parental responsiveness at T1 and students’ test result at T2 did not show a significant relationship in the bivariate correlations. Therefore, more research with standardized achievement tests is needed to verify the negative link we found between parental responsiveness and test results at the elementary school level.

For the links in the opposite direction, that is, students’ academic functioning at T1 predicting parental responsiveness and structure at T2, we also found only limited evidence. Only one indicator of academic functioning, namely, homework procrastination, was linked to parental responsiveness and structure. Interestingly, this is in line with prior research on secondary school students by Dumont et al. (2014), who did not find an effect of students’ reading achievement on responsiveness and structure, whereas students’ homework behavior predicted both quality dimensions. Dumont et al. (2014) concluded that students’ homework behavior might be particularly important for positive forms of parental homework assistance. Because the homework situation is often perceived as a situation with the potential for conflict (Dumont et al., 2012), parents of children who often procrastinate in homework situations may become stressed or irritated by their children’s behavior and feel less inclined to assist with homework in the future.

#### 4.1.2. Links between parental control and students’ academic functioning (H1b)

For parental control, we found a reciprocal relationship with students’ mean grades. All other expected links – bidirectional as well as unidirectional – were not significant. These findings are interesting in several regards. First, the reciprocal link between parental control and mean grades is in line with prior research on unidirectional associations that found that parental control negatively predicted students’ grades at both the elementary and the secondary level (Moroni et al., 2015; Núñez et al., 2015) and that, conversely, students’ achievement predicted parental controlling or monitoring behavior (e.g., Silinskas et al., 2013; Dumont et al., 2014; Núñez et al., 2017). Following Dumont et al. (2014), reciprocal relationships of control are alarming because students and parents might get into a vicious circle of increasing control and simultaneously decreasing academic functioning. This vicious circle might be particularly harmful for low achievers because they have been found to be more sensitive to controlling behavior (Ng et al., 2004; Grolnick and Pomerantz, 2022). In contrast to mean grades, no links were found between parental control and the other achievement variable, students’ test results, (in either direction). This differs from research on secondary school students, which has shown that parental control negatively predicted the results of standardized reading tests (Moroni et al., 2015), but it is in line with prior research on elementary school students that also did not find a link between controlling behavior and standardized test results (Silinskas et al., 2013; Wu et al., 2022). One possible explanation for the missing link is that grades are more salient and meaningful for parents than the results of standardized achievement tests. Parents receive a considerable amount of information on their children’s performance levels in the form of grades, but they often have little information on children’s standing in objective achievement tests. Moreover, grades are key determinants of children’s educational careers and, thus, are of high relevance for parents.

The missing link between prior parental control and students’ subsequent homework behavior is in contrast to prior research on secondary school students that found unfavorable effects of parental control on students’ homework behavior (Trautwein et al., 2006; Dumont et al., 2012; Moroni et al., 2015; Guill et al., 2020), but it is in line with prior research on elementary school students (Núñez et al., 2015). This lack of associations for elementary school students might be explained by younger students’ less pronounced need for autonomy and their positive attitude towards school and towards their parents, which might be relatively robust to external influences such as parents’ controlling behavior (Eccles et al., 1993; Hoover-Dempsey and Sandler, 1995). Regarding students’ homework behavior as a predictor of parental control, our finding that students’ unfavorable homework behavior did not result in more control is again in line with the findings of Dumont et al. (2014) on secondary school students. They argued that unfavorable homework behavior might be less alarming for parents than low grades and, therefore, only leads to decreases in positive forms of parental homework assistance (i.e., responsiveness and structure, see H1a) and not to increases in controlling behavior.

To sum up, we were able to apply the quality dimensions of homework assistance derived from the SDT (Ryan and Deci, 2000) to students at elementary school. However, our results suggest that findings on the effects of the quality dimensions on students’ academic functioning from prior research on secondary students cannot be simply transferred to elementary students. Therefore, more research that compares different age groups is needed. Regarding the effects of students’ academic functioning on the quality dimensions, the pattern we found for elementary school students is similar to the pattern that was found by Dumont et al. (2014) for older students. However, more research is needed to verify these results.

### 4.2. Scholastic homework assistance

The second aim of our study was to test whether there are reciprocal relationships between the quality of scholastic homework assistance and students’ academic functioning. The reason we investigated this is the high relevance of homework assistance at school and the lack of quantitative research on the quality of homework assistance provided in the scholastic environment.

#### 4.2.1. Links between scholastic responsiveness, structure, and students’ academic functioning (H2a)

Similar to the results on parental homework assistance found in our study and in contrast to the results of research on parental homework assistance for older students (Dumont et al., 2014; Moroni et al., 2015; Guill et al., 2020), we did not find that scholastic responsiveness and structure predicted students’ academic functioning. The lack of links found for structure is especially surprising because prior research on secondary school students has found positive effects when structuring homework assistance is given by persons other than the parents, namely, private tutors (Guill et al., 2020). However, similar to our considerations regarding the comparable results for parental homework assistance, due to students’ young age and their higher need for support, there might be too little variance in the responsiveness and structure provided by homework assistants at school to result in significant relationships. Moreover, one could assume that scholastic homework assistants, who are often pedagogical staff, show high mean levels of responsiveness and structure due to their professional knowledge. However, the surprising finding that structure was lower in the scholastic than in the parental setting could be because, at school, it is harder to achieve high levels of structure (for example, a quiet learning environment) than at home because homework assistance at school mostly takes place in a group setting.

Regarding our finding that scholastic responsiveness was positively predicted by students’ mean grades, it is possible that students with better grades have other characteristics that favor more responsiveness that we did not control for, for example, more socially adjusted behavior that might make it easier for them to ask for help in an appropriate way. Moreover, it is possible that students with better grades rate their homework environment more positively as they might have a more positive attitude to school and homework in general or might be able to make better use of the support provided by the homework assistants. Still, this finding is alarming because it points to potential inequalities in educational support, which may further increase existing differences.

The missing link between students’ prior procrastination and subsequent scholastic responsiveness and structure is one major difference between our results and prior results on parental homework assistance. Possibly, supervisors at school are less emotionally involved when their students procrastinate because students’ homework behavior might be less personally important to them than it is to the students’ parents.

#### 4.2.2. Links between scholastic control and students’ academic functioning (H2b)

Unlike our findings for parental homework assistance, no links were found between prior scholastic control and students’ subsequent academic achievement. One possible explanation for this is that students received scholastic homework assistance in addition to parental homework assistance (45.1% of the students at T1 and 38.4% of the students at T2 stated that they received scholastic homework assistance only one to two times per week). Thus, for students who receive scholastic homework assistance less often, the level of scholastic control might play a smaller role than the level of parental control. Therefore, future research should investigate a (sub-)sample consisting of students who receive homework assistance predominantly at school.

The missing link between students’ academic achievement at T1 and scholastic control at T2 is particularly surprising because this association has been consistently found in prior research on parental homework assistance for both elementary and secondary school students (e.g., Silinskas et al., 2013; Dumont et al., 2014; Núñez et al., 2017) and we also found this link for parental homework assistance in the present study. However, it has been discussed that controlling behavior might stem from pressure that is related to parental involvement. This pressure can come from multiple sources, for example, a child’s low achievement or parents’ or others’ expectations regarding the child’s performance (Dumont et al., 2014; Grolnick and Pomerantz, 2022). Compared to parents, homework assistants at school might feel less pressure if students have low academic achievement because they are more distanced from the students’ school career.

However, we found that students reported more procrastination the more control they perceived during scholastic homework assistance, which is in line with prior research on parental homework assistance (Dumont et al., 2014) and homework assistance given by both parents and private tutors (Dumont et al., 2014; Guill et al., 2020).

Taken together, we were able to apply the quality dimensions of homework assistance derived from the SDT (Ryan and Deci, 2000) to the context of scholastic homework assistance. However, only few of the relationships we had expected to find were statistically significant. On a descriptive level, one common finding for parental and scholastic homework assistance was that responsiveness and structure did not predict students’ academic functioning. However, control was related with students’ outcomes in both environments, but with different indicators of their academic functioning. Another difference between the two homework environments was that the two positive forms of homework assistance (i.e., responsiveness and structure) were predicted by different indicators of academic functioning. However, more research is needed to verify these commonalities and differences between the two homework environments.

### 4.3. Strengths and limitations

Our study has several strengths. First, we investigated homework assistance provided for elementary school students; this population has been studied less than other populations and previous results obtained for this population have been inconclusive. Second, we investigated homework assistance at school, which has not yet been addressed in quantitative studies. Third, we measured the quality of homework assistance instead of its quantity, which has been shown to be the more important predictor, and we included structure as a quality dimension that has been less studied up until now, although it has been argued that it distinguishes positive types of controlling behavior from intrusive ones (Grolnick and Pomerantz, 2009; Dumont et al., 2014; Guill et al., 2020). Fourth, we considered both grades and a test result to measure students’ academic achievement. Finally, the major strength of our study is the longitudinal design, which allowed us to control for the prior quality of homework assistance and students’ prior indicators of academic functioning.

However, there are also some limitations that should be considered when interpreting our results. First of all, we were not able to control for students being nested in classes because of the combination of the relatively small number of classes in our sample and the complexity of the analytical models (i.e., the number of parameters that need to be estimated in SEM). It might be important to control for clustering in classes because there might be class effects due to differences in the quality of homework assignments (Trautwein et al., 2006) that might influence students’ achievement and their homework behavior by being more or less interesting and activating. Moreover, the subsample for scholastic homework assistance was relatively small, which might have made it less likely to yield statistically significant and reliable results. Although the scales measuring the quality of homework assistance showed good reliability in prior studies at the secondary school level (Dumont et al., 2014; Guill et al., 2020), some of the scales showed rather low reliability in our sample (e.g., parental responsiveness at T1, with a value of α = 0.55). Another limitation was that, following the criteria proposed by Cheung and Rensvold (2002), metric measurement invariance and conventional norms for acceptable fit were violated for some constructs (i.e., parental control and students’ procrastination). Although Robitzsch and Lüdtke (2022) recently questioned whether measurement invariance is a prerequisite for group comparisons and comparisons across time points, it cannot be ruled out that different psychometric properties of the measurements across the two time points might have impeded the accuracy of our results (Cheung and Rensvold, 2002). Moreover, although we controlled for measurement errors, the stabilities of the constructs, and a considerable number of relevant covariates, there might be other unobserved confounders (e.g., students’ self-regulation, which might influence both the quality of parental homework assistance and students’ outcomes) that could lead to distorted estimates of cross-lagged effects in cross-lagged panel designs (Lüdtke and Robitzsch, 2022).

Additionally, there are some limitations regarding the assessment of the constructs. It might be productive to assess the quality of parental homework assistance and students’ homework behavior subject-specifically. Regarding the quality of homework assistance, Wild and Gerber (2007) argued that the quality might depend on students’ performance, which differs between the subjects, and on parents’ subject-specific perception of their own competence. Students’ homework behavior might also be subject-specific because of subject-specific differences in students’ competencies, interest, and intrinsic motivation (Trautwein et al., 2006). Finally, we relied solely on student reports although it might be more informative to additionally use reports from multiple sources, for example, from parents, teachers, or observers (Grolnick and Slowiaczek, 1994; Hoover-Dempsey et al., 2005).

### 4.4. Theoretical, practical, and scientific implications

We were able to apply the quality dimensions of parental homework assistance derived from the SDT (Ryan and Deci, 2000) to elementary school students and to homework assistance that is given at school. Theoretically, this supports the assumption that the SDT is generally valid across developmental stages and different learning environments. However, our findings differed from the findings of prior research on secondary school students and on homework assistance given by parents. This provides first indications that the quality dimensions might affect students’ outcomes differently at different developmental stages, which is in line with the theoretical assumptions of Eccles et al. (1993) and Hoover-Dempsey and Sandler (1995) that students benefit most if their educational environment matches their developmental needs. Taken together, when measuring the quality of homework assistance on the basis of the satisfaction of students’ needs, it might be beneficial to use an approach that keeps in mind that students’ needs change over the course of their development.

Despite the limitations of our study, practical implications can be derived from its results. Because control was negatively related with students’ academic functioning in both environments, both parents and homework assistants at school should be informed about the negative consequences of controlling and intrusive behavior for students’ academic functioning. Interventions and training for parents on homework assistance seem to be a promising approach as Patall et al. (2008) as well as Wild and Gerber (2009) reported encouraging effects. As homework assistance at school is becoming more and more important, it might be promising to also offer these interventions and this training for homework assistants at schools. It has been argued that low-quality homework assistance partly stems from pressure that is related to parental involvement (Dumont et al., 2014; Grolnick and Pomerantz, 2022). Therefore, reducing pressure might be a promising approach to decrease the use of control during homework assistance and to break the vicious circle between control and low academic achievement. This might be achieved by creating positive school climates for parents or by supporting effective school-parent communication (Grolnick and Pomerantz, 2022). Moreover, homework assistants at schools should be sensitized to the importance of being equally responsive to students regardless of their academic achievement. One promising approach to increase homework assistants’ awareness of and responsiveness to students’ needs could be a mindfulness-based professional development program that has shown encouraging findings in increasing teachers’ social and emotional competence and classroom interactions (Jennings et al., 2017). Although we did not find positive relationships of prior parental responsiveness and structure with students’ subsequent academic functioning, we still argue that parents should continue to provide responsiveness and structure even if their child shows unfavorable homework behavior because – following the SDT (Ryan and Deci, 2000) – these positive forms of homework assistance have the potential to support students’ needs and, although there has not been enough research on younger students, there have been promising findings for older students (e.g., Dumont et al., 2014; Moroni et al., 2015; Guill et al., 2020). Moreover, because responsiveness and structure have been found to be relevant for secondary school students, it makes sense that parents already begin to establish and maintain positive forms of homework assistance in the early years of schooling.

Finally, scientific implications for future research can be derived from our study. Because we found partly different results for elementary school students to those found in prior research on secondary school students, future research should compare the relationships for students from different age groups. Therefore, future research should follow Núñez et al. (2015) and compare the links found between the quality of homework assistance and students’ outcomes in different age groups or should use longitudinal data with longer time periods to determine which types of homework assistance are suitable for which age group. Moreover, more research on different homework settings and research that compares these settings is needed to verify our results. Due to the expansion of full-day schools in Germany, future research should pay special attention to scholastic homework assistance. In this context, it would be valuable to distinguish between different providers of homework assistance (e.g., pedagogical staff, teachers, or university students) who have heterogeneous qualifications and, thus, are likely to differ in the quality of the homework assistance they provide. For example, homework assistance can be provided by pedagogical staff who can be assumed to be aware of the benefits of need-supportive behavior or by university students who are not yet fully trained and might have less expertise (Guill et al., 2020). Finally, it needs to be considered that the expansion of full-day schools has raised questions about the role of homework and that some full-day schools have developed substitute or complementary programs for homework. For example, Brisson and Theis (2020) reported that study periods that were integrated into compulsory education had benefits compared to traditional homework regarding task quality, perceived cost and competence, and students’ well-being.

## 5. Conclusion

Whereas prior research has relatively consistently shown that there are reciprocal relationships between the quality of parental homework assistance and students’ academic functioning at secondary school, less is known about links between the two constructs for elementary school students and for homework assistance that is given at school. The present study therefore adds to the body of literature on homework assistance and its links with students’ outcomes. Taken together, we found fewer associations than expected based on the SDT (Ryan and Deci, 2000) and on the findings from prior research on secondary school students. Therefore, our results show that findings from prior research cannot simply be transferred to other age groups and other homework settings. Moreover, our findings are complex because they differed between the quality dimensions, the indicators of academic functioning, and the two settings that we investigated. Therefore, more research is needed that compares the associations between homework assistance and students’ academic functioning for different age groups, including students at elementary school, and in different homework settings.

## Data availability statement

The raw data supporting the conclusions of this article will be made available by the authors, without undue reservation.

## Ethics statement

The studies involving human participants were reviewed and approved by Ministry of Education, Science, Research and Culture in Schleswig-Holstein. Written informed consent to participate in this study was provided by the participants’ legal guardian/next of kin.

## Author contributions

LB was responsible for the data analysis and interpretation, for the literature search, and for writing the manuscript. KK made important contributions to the structure and content of the manuscript. OL made important contributions to the data analysis strategy. KG designed the study and supervised the data collection. KK, JR, OL, and KG provided important input on how to improve the manuscript draft. All authors contributed to the article and approved the submitted version.

## Conflict of interest

The authors declare that the research was conducted in the absence of any commercial or financial relationships that could be construed as a potential conflict of interest.

## Publisher’s note

All claims expressed in this article are solely those of the authors and do not necessarily represent those of their affiliated organizations, or those of the publisher, the editors and the reviewers. Any product that may be evaluated in this article, or claim that may be made by its manufacturer, is not guaranteed or endorsed by the publisher.

## Footnotes

^1^In Germany, there are three types of full-day schools. The difference between the three types lies in whether the full-day program is compulsory for all students (*gebundene Ganztagsschule*), for just some students (*teilgebundene Ganztagsschule*), or is an optional addition to compulsory half-day school (*offene Ganzstagsschule*). Optional full-day programs are the most common type (StEG-Konsortium, 2019).

^2^There was no option for 1+, 5+, 5–, 6+, and 6– because these grades are rarely given.
