# Supplementary material for: Reciprocal relationships between parental and scholastic homework assistance and students’ academic functioning at elementary school
Source: Front Psychol. 2023 Apr 28;14:1106362. doi: 10.3389/fpsyg.2023.1106362 (PMC10231229; doi:10.3389/fpsyg.2023.1106362)
Supplement: Supplementary file 1 [file Table_1.DOCX]

Supplementary Material

# Supplementary Tables

# Table S1

*List of items measuring students’ homework behavior and the quality of parental and scholastic homework assistance*

| *Scale* | *Items* | |
| --- | --- | --- |
| *Homework effort*  (adapted from Trautwein et al., 2006) |  | |
| (1) | Please think about the homework you got in the last two weeks. How many of them did you do as well as you could? | |
| (2) | I’m really diligent about homework. | |
| (3) | I do my homework as best as I can. | |
| (4) | I really make an effort with my homework. | |
| (5) | I do all my homework very neatly. | |
| *Homework procrastination*  (Dumont et al., 2014) |  | |
| (1) | It often takes me a long time to get going with my homework. | |
| (2) | I put off doing my homework until the last minute. | |
| (3) | I put off starting my homework for so long that I don’t finish it on time. | |
| *Quality of parental homework assistance* (Dumont et al., 2014) |  | |
| *Responsiveness* |  | |
| (1) | My parents help me with my homework if I ask them to. | |
| (2) | My parents always help me with my homework when I’m having difficulties with it. | |
| (3) | When I’m doing my homework, I can ask my parents for help at any time. | |
| (4) | When I’m doing my homework, my parents carefully listen to how I would complete the task instead of telling me what to do straight away. | |
| *Structure* |  | |
| (1) | My parents want me to do my homework first before I meet friends. | |
| (2) | It is important to my parents that I have enough time to do my homework. | |
| (3) | My parents make sure that I do my homework in a quiet environment where I am not disturbed by, for example, music, TV, or phone calls. | |
| (4) | My parents have explained to me why it is important to do my homework at a desk and not, for instance, in front  of the TV. | |
| (5) | My parents help me so that I have everything I need (e.g., ruler, pens, etc.) when I do my homework. | |
| (6) | My parents make sure that I have enough time and space to do my homework. | |
| *Control* |  | |
| (1) | My parents sometimes help me with my homework even when I don’t need any help. | |
| (2) | My parents often interfere when I’m doing my homework. | |
| (3) | My parents sit next to me when I’m doing homework and immediately correct any mistakes I make. | |
| (4) | My parents threaten to punish me (e.g., TV ban) if I don’t work hard enough on my homework. | |
| *Quality of scholastic homework assistance*  (adapted from Dumont et al., 2014) |  | |
| (1) | My supervisor helps me with my homework if I ask them to. | |
| (2) | My supervisor always helps me with my homework when I’m having difficulties with it. | |
| (3) | When I’m doing my homework, I can ask my supervisor for help at any time. | |
| (4) | When I’m doing my homework, my supervisor carefully listens to how I would complete the task instead of telling me what to do straight away. | |
| *Structure* |  | |
| (1) | It is important to my supervisor that I have enough time to do my homework. | |
| (2) | My supervisor makes sure that I do my homework in a quiet environment where I am not disturbed by, for example, music or mobile phones. | |
| (3) | My supervisor helps me so that I have everything I need (e.g., ruler, pens, etc.) when I do my homework. | |
| (4) | My supervisor makes sure that I have enough time and space to do my homework. | |
| *Control* |  |  |
| (1) | My supervisor sometimes helps me with my homework even when I don’t need any help. | |
| (2) | My supervisor often interferes when I’m doing my homework. | |
| (3) | My supervisor sits next to me when I’m doing homework and immediately corrects any mistakes I make. | |
| (4) | My supervisor gets angry if I don’t work hard enough on my homework. | |

**Table S2**

*Results of the cross-lagged models: Links between parental responsiveness and the four indicators of students’ academic functioning*

|  | Model 1 (*N* = 319^a^) | | | | Model 2 (*N* = 335) | | | | | Model 3 (*N* = 319^a^) | | | | | Model 4 (*N* = 319^a^) | | | | |
| --- | --- | --- | --- | --- | --- | --- | --- | --- | --- | --- | --- | --- | --- | --- | --- | --- | --- | --- | --- |
|  | Mean grade T2 | | Responsive-ness T2 | | | Test result T2 | | Responsive-ness T2 | | | Effort T2 | | Responsive-ness T2 | | | Procrastina-tion T2 | | Responsive-ness T2 | |
|  | β | *SE* | β | *SE* | | β | *SE* | β | *SE* | | β | *SE* | β | *SE* | | β | *SE* | β | *SE* |
| Responsiveness T1 | –.04 | .07 | **.46** | .08 | | –**.13** | .07 | **.47** | .08 | | –.10 | .08 | **.46** | .08 | | .07 | .09 | **.46** | .08 |
| Mean grade T1 | **.61** | .05 | .03 | .07 | | - | - | - | - | | - | - | - | - | | - | - | - | - |
| Test result T1 | - | - | - | - | | **.61** | .04 | .00 | .07 | | - | - | - | - | | - | - | - | - |
| Effort T1 | - | - | - | - | | - | - | - | - | | **.63** | .06 | .03 | .08 | | - | - | - | - |
| Procrastination T1 | - | - | - | - | | - | - | - | - | | - | - | - | - | | **.49** | .08 | –**.22** | .08 |
| *Covariates* |  |  |  |  | |  |  |  |  | |  |  |  |  | |  |  |  |  |
| Gender^b^ | .03 | .06 | .11 | .06 | | –.05 | .05 | .12 | .06 | | .07 | .06 | .11 | .06 | | .00 | .07 | .10 | .06 |
| Migration background^c^ | .02 | .06 | **.20** | .07 | | –.04 | .06 | **.19** | .07 | | .02 | .07 | **.20** | .07 | | –.01 | .08 | **.20** | .07 |
| Educational background^d^ | –.07 | .10 | .09 | .11 | | –.08 | .09 | .10 | .12 | | –.05 | .10 | .10 | .11 | | –.11 | .12 | .12 | .11 |
| Educational background^e^ | –.11 | .09 | .12 | .11 | | –.12 | .09 | .13 | .11 | | .02 | .10 | .13 | .11 | | –.17 | .12 | 0.17 | .11 |
| *Model Fit* |  |  |  |  | |  |  |  |  | |  |  |  |  | |  |  |  |  |
| *R*² | .39 | | .33 | | | .39 | | .33 | | | .42 | | .33 | | | .24 | | .38 | |
| *X*² | 95.35 | | | | | 81.89 | | | | | 283.70 | | | | | 178.18 | | | |
| df | 54 | | | | | 54 | | | | | 183 | | | | | 109 | | | |
| CFI | .93 | | | | | .95 | | | | | .94 | | | | | .93 | | | |
| TLI | .89 | | | | | .93 | | | | | .92 | | | | | .90 | | | |
| RMSEA | .05 | | | | | .04 | | | | | .04 | | | | | .05 | | | |
| SRMR | .05 | | | | | .05 | | | | | .06 | | | | | .06 | | | |

*Note*. Standardized regression coefficients (continuous variables were stdyx- and binary variables were stdy-standardized). Significant coefficients are shown in bold (*p* ≤ .05). T1/T2 = first/second point of measurement, CFI = comparative fit index, TLI = Tucker–Lewis index, RMSEA = root mean square error of approximation, SRMR = standardized root mean square residual. ^a^The reduced sample size is due to students who had missing values on all variables. Reference categories: ^b^male, ^c^language other than German spoken at home, ^d^neither parent qualified for university/student did not know, ^e^neither parent qualified for university/at least one parent qualified for university.

**Table S3**

*Results of the cross-lagged models: Links between parental structure and the four indicators of students’ academic functioning*

|  | Model 5 (*N* = 319^a^) | | | | | | Model 6 (*N* = 335) | | | | | | Model 7 (*N* = 319^a^) | | | | | | Model 8 (*N* = 319^a^) | | | | | |
| --- | --- | --- | --- | --- | --- | --- | --- | --- | --- | --- | --- | --- | --- | --- | --- | --- | --- | --- | --- | --- | --- | --- | --- | --- |
|  | Mean grade T2 | | | Structure T2 | | | Test result T2 | | | Structure T2 | | | Effort T2 | | | Structure T2 | | | Procrastina-tion T2 | | | Structure T2 | | |
|  | β | *SE* | β | | *SE* | β | | *SE* | β | | *SE* | β | | *SE* | β | | *SE* | β | | *SE* | β | | *SE* |  |
| Structure T1 | –.03 | .07 | **.59** | | .08 | .09 | | .07 | **.60** | | .07 | –.01 | | .08 | **.56** | | .08 | –.05 | | .09 | **.59** | | .07 |  |
| Mean grade T1 | **.61** | .05 | .01 | | .08 | - | | - | - | | - | - | | - | - | | - | - | | - | - | | - |  |
| Test result T1 | - | - | - | | - | **.59** | | .04 | .02 | | .07 | - | | - | - | | - | - | | - | - | | - |  |
| Effort T1 | - | - | - | | - | - | | - | - | | - | **.62** | | .06 | .10 | | .08 | - | | - | - | | - |  |
| Procrastination T1 | - | - | - | | - | - | | - | - | | - | - | | - | - | | - | **.48** | | .08 | **–.18** | | .08 |  |
| *Covariates* |  | |  | | |  | | |  | | |  | | |  | | |  | | |  | | |  |
| Gender^b^ | .04 | .06 | .02 | | .07 | –.07 | | .05 | .03 | | .07 | .07 | | .06 | .00 | | .07 | .01 | | .07 | .01 | | .07 |  |
| Migration background^c^ | .02 | .06 | –.11 | | .08 | –.08 | | .06 | –.12 | | .08 | .02 | | .07 | –.11 | | .08 | –.01 | | .08 | –.10 | | .08 |  |
| Educational background^d^ | –.07 | .10 | –.14 | | .12 | –.09 | | .09 | –.15 | | .12 | –.06 | | .10 | –.14 | | .12 | –.10 | | .12 | –.12 | | .11 |  |
| Educational background^e^ | –.12 | .09 | –.21 | | .12 | –.10 | | .09 | –.21 | | .12 | .00 | | .10 | –.22 | | 12 | –.17 | | .12 | –.18 | | .12 |  |
| *Model Fit* |  | |  | | |  | | |  | | |  | | |  | | |  | | |  | | |  |
| *R*² | .39 | | .40 | | | .38 | | | .41 | | | .41 | | | .41 | | | .24 | | | .43 | | |  |
| *X*² | 155.28 | | | | | 139.66 | | | | | | 365.13 | | | | | | 275.14 | | | | | |  |
| df | 112 | | | | | 107 | | | | | | 273 | | | | | | 183 | | | | | |  |
| CFI | .93 | | | | | .95 | | | | | | .94 | | | | | | .91 | | | | | |  |
| TLI | .91 | | | | | .93 | | | | | | .93 | | | | | | .89 | | | | | |  |
| RMSEA | .04 | | | | | .03 | | | | | | .03 | | | | | | .04 | | | | | |  |
| SRMR | .05 | | | | | .05 | | | | | | .05 | | | | | | .06 | | | | | |  |

*Note*. Standardized regression coefficients (continuous variables were stdyx- and binary variables were stdy-standardized). Significant coefficients are shown in bold (*p* ≤ .05). T1/T2 = first/second point of measurement, CFI = comparative fit index, TLI = Tucker–Lewis index, RMSEA = root mean square error of approximation, SRMR = standardized root mean square residual. ^a^The reduced sample size is due to students who had missing values on all variables. Reference categories: ^b^male, ^c^language other than German spoken at home, ^d^neither parent qualified for university/student did not know, ^e^neither parent qualified for university/at least one parent qualified for university.

**Table S4**

*Results of the cross-lagged models: Links between parental control and the four indicators of students’ academic functioning*

|  | Model 9 (*N* = 319^a^) | | | | Model 10 (*N* = 335) | | | | | Model 11 (*N* = 319^a^) | | | | Model 12 (*N* = 319^a^) | | | |
| --- | --- | --- | --- | --- | --- | --- | --- | --- | --- | --- | --- | --- | --- | --- | --- | --- | --- |
|  | Mean grade T2 | | Control T2 | | Test result T2 | | | Control T2 | | Effort T2 | | Control T2 | | Procrastina-tion T2 | | Control T2 | |
|  | β | *SE* | β | *SE* | β | *SE* | β | | *SE* | β | *SE* | β | *SE* | β | *SE* | β | *SE* |
| Control T1 | **–.22** | .07 | **.46** | .09 | –.06 | .07 | **.48** | | .08 | .00 | .08 | **.48** | .08 | .14 | .10 | **.53** | .10 |
| Mean grade T1 | **.55** | .05 | **–.16** | .08 | - | - | - | | - | - | - | - | - | - | - | - | - |
| Test result T1 | - | - | - | - | **.59** | .04 | –.09 | | .07 | - | - | - | - | - | - | - | - |
| Effort T1 | - | - | - | - | - | - | - | | - | **.61** | .06 | –.02 | .08 | - | - | - | - |
| Procrastination T1 | - | - | - | - | - | - | - | | - | - | - | - | - | **.43** | .10 | –.12 | .10 |
| *Covariates* |  |  |  |  |  |  |  | |  |  |  |  |  |  |  |  |  |
| Gender^b^ | .05 | .06 | **–.14** | .07 | –.07 | .05 | **–.17** | | .07 | .07 | .06 | **–.16** | .07 | **–**.01 | .07 | **–.17** | .07 |
| Migration background^c^ | –.01 | .06 | –.10 | .08 | –.08 | .06 | –.12 | | .08 | .01 | .07 | –.11 | .08 | .02 | .08 | –.10 | .08 |
| Educational background^d^ | –.02 | .09 | .07 | .12 | –.07 | .09 | .06 | | .12 | –.07 | .10 | .04 | .12 | –.12 | .12 | .04 | .12 |
| Educational background^e^ | –.08 | .09 | .04 | .12 | –.10 | .09 | .05 | | .12 | .01 | .10 | .03 | .12 | –.18 | .12 | .04 | .12 |
| *Model Fit* |  |  |  |  |  |  |  | |  |  |  |  |  |  |  |  |  |
| *R*² | .43 | | .34 | | .38 | | .32 | | | .41 | | .30 | | .25 | | .31 | |
| *X*² | 116.55 | | | | 109.72 | | | | | 286.71 | | | | 198.55 | | | |
| df | 54 | | | | 54 | | | | | 183 | | | | 109 | | | |
| CFI | .88 | | | | .90 | | | | | .93 | | | | .90 | | | |
| TLI | .83 | | | | .84 | | | | | .91 | | | | .86 | | | |
| RMSEA | .06 | | | | .06 | | | | | .04 | | | | .05 | | | |
| SRMR | .06 | | | | .05 | | | | | .06 | | | | .06 | | | |

*Note*. Standardized regression coefficients (continuous variables were stdyx- and binary variables were stdy-standardized). Significant coefficients are shown in bold (*p* ≤ .05). T1/T2 = first/second point of measurement, CFI = comparative fit index, TLI = Tucker–Lewis index, RMSEA = root mean square error of approximation, SRMR = standardized root mean square residual. ^a^The reduced sample size is due to students who had missing values on all variables. Reference categories: ^b^male, ^c^language other than German spoken at home, ^d^neither parent qualified for university/student did not know, ^e^neither parent qualified for university/at least one parent qualified for university.

**Table S5**

*Results of the cross-lagged models: Links between scholastic responsiveness and the four indicators of students’ academic functioning*

|  | Model 13 (*N* = 112) | | | | Model 14 (*N* = 112) | | | | Model 15 (*N* = 112) | | | | Model 16 (*N* = 112) | | | |
| --- | --- | --- | --- | --- | --- | --- | --- | --- | --- | --- | --- | --- | --- | --- | --- | --- |
|  | Mean grade T2 | | Responsive-ness T2 | | Test result T2 | | Responsive-ness T2 | | Effort T2 | | Responsive-ness T2 | | Procrastina-tion T2 | | Responsive-ness T2 | |
|  | β | *SE* | β | *SE* | β | *SE* | β | *SE* | β | *SE* | β | *SE* | β | *SE* | β | *SE* |
| Responsiveness T1 | .12 | .14 | .41 | .30 | .16 | .13 | **.60** | .28 | –.12 | .15 | **.61** | .28 | –.12 | .20 | **.62** | .26 |
| Mean grade T1 | **.63** | .07 | **.33** | .15 | - | - | - | - | - | - | - | - | - | - | - | - |
| Test result T1 | - | - | - | - | **.57** | .08 | –.07 | .14 | - | - | - | - | - | - | - | - |
| Effort T1 | - | - | - | - | - | - | - | - | **.80** | .09 | –.01 | .22 | - | - | - | - |
| Procrastination T1 | - | - | - | - | - | - | - | - | - | - | - | - | **.41** | .13 | .15 | .18 |
| *Covariates* |  |  |  |  |  |  |  |  |  |  |  |  |  |  |  |  |
| Gender^a^ | .07 | .09 | .25 | .15 | **–.19** | .09 | .13 | .18 | .11 | .10 | .21 | .16 | –.16 | .12 | .18 | .16 |
| Migration background^b^ | –.07 | .09 | –.28 | .14 | .10 | .10 | –.20 | .15 | .18 | .11 | –.17 | .15 | –.05 | .14 | –.23 | .16 |
| Educational background^c^ | –.01 | .14 | **–.45** | .21 | –.21 | .13 | –.43 | .23 | .08 | .15 | **–.45** | .23 | –.08 | .19 | **–.44** | .22 |
| Educational background^d^ | –.09 | .13 | –.04 | .20 | **–.25** | .13 | –.06 | .22 | –.02 | .15 | –.07 | .21 | –.05 | .18 | –.09 | .22 |
| *Model Fit* |  |  |  |  |  |  |  |  |  |  |  |  |  |  |  |  |
| *R*² | .48 | | .51 | | .42 | | .45 | | .68 | | .48 | | .25 | | .50 | |
| *X*² | .00 | | | | .00 | | | | .00 | | | | .00 | | | |
| df | 0 | | | | 0 | | | | 0 | | | | 0 | | | |
| CFI | 1 | | | | 1 | | | | 1 | | | | 1 | | | |
| TLI | 1 | | | | 1 | | | | 1 | | | | 1 | | | |
| RMSEA | .00 | | | | .00 | | | | .00 | | | | .00 | | | |
| SRMR | .00 | | | | .00 | | | | .00 | | | | .00 | | | |

*Note*. Standardized regression coefficients (continuous variables were stdyx- and binary variables were stdy-standardized). Significant coefficients are shown in bold (*p* ≤ .05). T1/T2 = first/second point of measurement, CFI = comparative fit index, TLI = Tucker–Lewis index, RMSEA = root mean square error of approximation, SRMR = standardized root mean square residual.

Reference categories: ^a^male, ^b^language other than German spoken at home, ^c^neither parent qualified for university/student did not know, ^d^neither parent qualified for university/at least one parent qualified for university.

**Table S6**

*Results of the cross-lagged models: Links between scholastic structure and the four indicators of students’ academic functioning*

|  | Model 17 (*N* = 112) | | | | | Model 18 (*N* = 112) | | | | Model 19 (*N* = 112) | | | | Model 20 (*N* = 112) | | | |
| --- | --- | --- | --- | --- | --- | --- | --- | --- | --- | --- | --- | --- | --- | --- | --- | --- | --- |
|  | Mean grade T2 | | Structure T2 | | | Test result T2 | | Structure T2 | | Effort T2 | | Structure T2 | | Procrastina-tion T2 | | Structure T2 | |
|  | β | *SE* | β | *SE* | β | | *SE* | β | *SE* | β | *SE* | β | *SE* | β | *SE* | β | *SE* |
| Structure T1 | –.04 | .21 | **.64** | .23 | –.02 | | .12 | **.74** | .19 | –.12 | .15 | **.59** | .23 | –.21 | .19 | .71 | .21 |
| Mean grade T1 | **.64** | .08 | .20 | .17 | - | | - | - | - | - | - | - | - | - | - | - | - |
| Test result T1 | - | - | - | - | **.60** | | .07 | .15 | .14 | - | - | - | - | - | - | - | - |
| Effort T1 | - | - | - | - | - | | - | - | - | **.79** | .08 | .15 | .19 | - | - | - | - |
| Procrastination T1 | - | - | - | - | - | | - | - | - | - | - | - | - | **.46** | .13 | –.09 | .17 |
| *Covariates* |  |  |  |  |  | |  |  |  |  |  |  |  |  |  |  |  |
| Gender^a^ | .09 | .09 | .25 | .15 | –.16 | | .08 | .25 | .15 | .09 | .10 | .25 | .15 | –.16 | .12 | .22 | .16 |
| Migration background^b^ | –.07 | .11 | –.26 | .17 | .08 | | .10 | –.21 | .17 | .14 | .12 | –.20 | .16 | –.10 | .15 | –.20 | .17 |
| Educational background^c^ | .02 | .14 | –.24 | .23 | –.16 | | .12 | –.23 | .22 | .03 | .14 | –.22 | .22 | –.09 | .17 | –.18 | .22 |
| Educational background^d^ | –.07 | .14 | –.01 | .22 | –.23 | | .13 | –.05 | .22 | –.06 | .14 | –.06 | .22 | –.08 | .18 | .01 | .22 |
| *Model Fit* |  |  |  |  |  | |  |  |  |  |  |  |  |  |  |  |  |
| *R*² | .46 | | .57 | | .41 | | | .68 | | .68 | | .53 | | .29 | | .63 | |
| *X*² | .00 | | | | .00 | | | | | .00 | | | | .00 | | | |
| df | 0 | | | | 0 | | | | | 0 | | | | 0 | | | |
| CFI | 1 | | | | 1 | | | | | 1 | | | | 1 | | | |
| TLI | 1 | | | | 1 | | | | | 1 | | | | 1 | | | |
| RMSEA | .00 | | | | .00 | | | | | .00 | | | | .00 | | | |
| SRMR | .00 | | | | .00 | | | | | .00 | | | | .00 | | | |

*Note*. Standardized regression coefficients (continuous variables were stdyx- and binary variables were stdy-standardized). Significant coefficients are shown in bold (*p* ≤ .05). T1/T2 = first/second point of measurement, CFI = comparative fit index, TLI = Tucker–Lewis index, RMSEA = root mean square error of approximation, SRMR = standardized root mean square residual.

Reference categories: ^a^male, ^b^language other than German spoken at home, ^c^neither parent qualified for university/student did not know, ^d^neither parent qualified for university/at least one parent qualified for university.

**Table S7**

*Results of the cross-lagged models: Links between scholastic control and the four indicators of students’ academic functioning*

|  | Model 21 (*N* = 112) | | | | Model 22 (*N* = 112) | | | | Model 23 (*N* = 112) | | | | Model 24 (*N* = 112) | | | |
| --- | --- | --- | --- | --- | --- | --- | --- | --- | --- | --- | --- | --- | --- | --- | --- | --- |
|  | Mean grade T2 | | Control T2 | | Test result T2 | | Control T2 | | Effort T2 | | Control T2 | | Procrastina-tion T2 | | Control T2 | |
|  | β | *SE* | β | *SE* | β | *SE* | β | *SE* | β | *SE* | β | *SE* | β | *SE* | β | *SE* |
| Control T1 | .09 | .14 | **.69** | .20 | .03 | .11 | **.64** | .17 | .18 | .13 | **.65** | .17 | **.47** | .20 | **.62** | .22 |
| Mean grade T1 | **.68** | .09 | .23 | .16 | - | - | - | - | - | - | - | - | - | - | - | - |
| Test result T1 | - | - | - | - | **.61** | .07 | –.02 | .13 | - | - | - | - | - | - | - | - |
| Effort T1 | - | - | - | - | - | - | - | - | **.79** | .08 | .03 | .16 | - | - | - | - |
| Procrastination T1 | - | - | - | - | - | - | - | - | - | - | - | - | .25 | .17 | .01 | .19 |
| *Covariates* |  |  |  |  |  |  |  |  |  |  |  |  |  |  |  |  |
| Gender^a^ | .09 | .09 | .13 | .13 | –.15 | .09 | .12 | .14 | .13 | .10 | .12 | .13 | –.07 | .12 | .11 | .13 |
| Migration background^b^ | –.04 | .10 | –.10 | .16 | .09 | .10 | –.06 | .16 | .22 | .12 | –.03 | .17 | .23 | .15 | –.08 | .18 |
| Educational background^c^ | .00 | .14 | –.10 | .21 | –.16 | .13 | –.01 | .20 | .00 | .14 | .02 | .20 | –.18 | .16 | –.02 | .19 |
| Educational background^d^ | –.08 | .13 | –.04 | .21 | –.23 | .13 | –.04 | .20 | –.07 | .14 | –.02 | .20 | –.16 | .17 | –.05 | .20 |
| *Model Fit* |  |  |  |  |  |  |  |  |  |  |  |  |  |  |  |  |
| *R*² | .46 | | .39 | | .40 | | .40 | | .68 | | .40 | | .40 | | .40 | |
| *X*² | .00 | | | | .00 | | | | .00 | | | | .00 | | | |
| df | 0 | | | | 0 | | | | 0 | | | | 0 | | | |
| CFI | 1 | | | | 1 | | | | 1 | | | | 1 | | | |
| TLI | 1 | | | | 1 | | | | 1 | | | | 1 | | | |
| RMSEA | .00 | | | | .00 | | | | .00 | | | | .00 | | | |
| SRMR | .00 | | | | .00 | | | | .00 | | | | .00 | | | |

*Note*. Standardized regression coefficients (continuous variables were stdyx- and binary variables were stdy-standardized). Significant coefficients are shown in bold (*p* ≤ .05). T1/T2 = first/second point of measurement, CFI = comparative fit index, TLI = Tucker–Lewis index, RMSEA = root mean square error of approximation, SRMR = standardized root mean square residual.

Reference categories: ^a^male, ^b^language other than German spoken at home, ^c^neither parent qualified for university/student did not know, ^d^neither parent qualified for university/at least one parent qualified for university.
